# Genomes of *Vibrio metoecus* co-isolated with *Vibrio cholerae* extend our understanding of differences between these closely related species

**DOI:** 10.1186/s13099-022-00516-x

**Published:** 2022-11-20

**Authors:** Fabini D. Orata, Nora A. S. Hussain, Kevin Y. H. Liang, Dalong Hu, Yann F. Boucher

**Affiliations:** 1 Department of Biological Sciences, University of Alberta, Edmonton, Alberta Canada; 2 Department of Chemical and Materials Engineering, University of Alberta, Edmonton, Alberta Canada; 3 Department of Quantitative Life Sciences, McGill University, Montréal, Québec Canada; 4 Lady Davis Institute, Jewish General Hospital, McGill University, Montréal, Québec Canada; 5 Saw Swee Hock School of Public Health, National University of Singapore and National University Hospital System, Singapore, Singapore; 6 Singapore Centre for Environmental Life Sciences Engineering, National University of Singapore, Singapore, Singapore; 7 Infectious Diseases Translational Research Program, Department of Microbiology and Immunology, Yong Loo Lin School of Medicine, National University of Singapore and National University Hospital System, Singapore, Singapore

**Keywords:** *Vibrio cholerae*, *Vibrio metoecus*, Comparative genomics, Horizontal gene transfer, Evolution

## Abstract

**Background:**

*Vibrio cholerae*, the causative agent of cholera, is a well-studied species, whereas *Vibrio metoecus* is a recently described close relative that is also associated with human infections. The availability of *V. metoecus* genomes provides further insight into its genetic differences from *V. cholerae*. Additionally, both species have been co-isolated from a cholera-free brackish coastal pond and have been suggested to interact with each other by horizontal gene transfer (HGT).

**Results:**

The genomes of 17 strains from each species were sequenced. All strains share a large core genome (2675 gene families) and very few genes are unique to each species (< 3% of the pan-genome of both species). This led to the identification of potential molecular markers—for nitrite reduction, as well as peptidase and rhodanese activities—to further distinguish *V. metoecus* from *V. cholerae*. Interspecies HGT events were inferred in 21% of the core genes and 45% of the accessory genes. A directional bias in gene transfer events was found in the core genome, where *V. metoecus* was a recipient of three times (75%) more genes from *V. cholerae* than it was a donor (25%).

**Conclusion:**

*V. metoecus* was misclassified as an atypical variant of *V. cholerae* due to their resemblance in a majority of biochemical characteristics. More distinguishing phenotypic assays can be developed based on the discovery of potential gene markers to avoid any future misclassifications. Furthermore, differences in relative abundance or seasonality were observed between the species and could contribute to the bias in directionality of HGT.

**Supplementary Information:**

The online version contains supplementary material available at 10.1186/s13099-022-00516-x.

## Background

*Vibrio cholerae* is the etiological agent of the potent diarrheal disease cholera, responsible for 1.2–4.3 million infections and 28,000–142,000 deaths worldwide every year [1]. All seven cholera pandemics throughout history were caused by either the Classical or El Tor biotype. Both biotypes were derived from a single genetic lineage, termed the pandemic generating lineage, within this extremely diverse species harboring more than 200 serogroups [2–4]. However, the majority of environmental *V. cholerae* isolates are actually non-toxigenic [5]. *V. cholerae* is a readily-culturable and genome-sequenced organism with close to 1700 whole-genome sequences available in the NCBI Microbial Genomes database [6] as of October 2022. Multiple comparative genomic studies have been performed with *V. cholerae* genomes to determine the population structure and genetic diversity of the species, with the focus mainly on clinical isolates [7–14]. Comparative genomics has been proven to be a useful tool in elucidating the tempo and mode of evolution of pathogenic *V. cholerae*, applied in the analyses of cholera outbreaks in Haiti in 2010 [7–10, 14] and Yemen in 2016 [11, 14], two of the largest cholera outbreaks in recent history.

By contrast, *Vibrio metoecus* is a recently described species and one of the closest known relatives of *V. cholerae* [15, 16] that remains poorly understood. It was initially described as an atypical variant of *V. cholerae* [17] as it appears as yellow *V. cholerae*-like cells on thiosulfate citrate bile sucrose (TCBS) agar; shares a high 16S rRNA gene sequence identity (> 98%) with *V. cholerae* reference genomes; and resembles *V. cholerae* in the majority of biochemical characteristics. However, it is negative for the production of acetoin (Voges-Proskauer assay), amylase, and lipase [15, 17, 18]. It was further determined that *V. metoecus* is able to grow using D-glucuronic acid or N-acetyl-D-galactosamine as sole carbon sources, both of which are distinguishing characteristics for the species [15]. So far, clinical and environmental strains had been isolated mostly in the USA [15, 18–20], and two environmental singletons had been isolated in Europe (Italy and Spain) [21, 22]. As of October 2022, genomes of only about 30 strains are available in the NCBI Microbial Genomes database [6], including the 12 sequenced in this study. Here, the availability of additional *V. metoecus* genomes provides more insight on the biology of the species and possible genetic differences from *V. cholerae* that can be useful in taxonomic identification to avoid misclassification.

Multiple strains of both *V. cholerae* and *V. metoecus* have been co-isolated from a cholera-free, brackish coastal pond in the US East Coast (Oyster Pond, Falmouth, Massachusetts, USA) [12, 13, 19]. The integron gene cassettes of geographically co-occurring *V. cholerae* and *V. metoecus* are more similar than geographically distinct *V. cholerae* (i.e., between isolates from the USA and Bangladesh) [19]. Thus, their co-isolation has led us to hypothesize that both species are likely in constant interaction with each other, providing opportunities for gene exchange by horizontal gene transfer (HGT). In this study, the genomes of 17 strains from each species originating from the same pond were screened for interspecies HGT events. Results suggest that up to three times as many genes were found to have moved from *V. cholerae* to *V. metoecus* than vice versa. From a previous study, it has been found that in this sampling site (Oyster Pond), *V. cholerae* is more abundant than *V. metoecus*, with an average of three times as many cells in a water sample [23]. *V. metoecus* environmental population has a strong seasonal bias in abundance, only being able to be significantly observed at the end of the summer (i.e., August to September) in Oyster Pond, while *V. cholerae* is always present throughout the entire summer (i.e., June to September) [23]. We hypothesize that these differences in relative abundance and/or seasonality could be contributing factors to the bias in HGT directionality from *V. cholerae* to *V. metoecus*.

## Methods

### Isolates of *V. cholerae* and *V. metoecus* used in this study

Environmental strains of *V. cholerae* and *V. metoecus* were isolated from Oyster Pond, Falmouth, Massachusetts, USA (41°32′31.8″N 70°38′19.9″W) on August 24 and September 19, 2009, as previously described [13]. In brief, three 100-L water samples were collected on each sampling date and sequentially filtered (63-, 5-, 1-, 0.22-μm filters). Filters were then placed on TCBS agar (Becton Dickinson) and incubated overnight at 37 °C. Yellow colonies (indicative of *Vibrio* species) were picked from the TCBS plates and streaked on tryptic soy agar (TSA) (Becton Dickinson) with 1.0% NaCl (BDH) and incubated overnight. Cultures in tryptic soy broth (TSB) (Becton Dickinson) were incubated overnight with shaking at 200 rpm. Preliminary identification of strains was subsequently performed by multilocus sequence analysis (MLSA) using seven housekeeping genes (*gppA*, *intI*, *mdh*, *mutS*, *pgi*, *plsX*, *recA*), as previously described [13].

### Genomic DNA extraction and sequencing

Whole-genome sequences were obtained from 17 strains each of *V. cholerae* and *V. metoecus* (Table 1). The genomes of seven strains (two *V. cholerae* and five *V. metoecus*) were sequenced previously [15, 20]. For the remaining 27 strains, genomic DNA was extracted from overnight TSB cultures using the DNeasy Blood and Tissue Kit (QIAGEN) and quantified using the Quant-iT PicoGreen dsDNA Assay Kit (Molecular Probes) and the Synergy H1 microplate reader (BioTek). A paired-end library was constructed using the Nextera XT DNA Library Preparation Kit (Illumina). Whole-genome sequencing was performed on the Illumina NextSeq platform using NextSeq 500/550 High Output Kit v2 (for 300 cycles), generating 150-bp paired-end reads. The CLC Genomics Workbench 7.5.2 [24] was used for raw read quality control and de novo assembly. Raw reads were first filtered using the following parameters: quality score limit = 0.05, maximum number of ambiguous nucleotides = 0, discard reads below length = 15. De novo assembly was then performed using the following parameters: word size = 45, bubble size = 98, minimum contig length = 1000, mismatch cost = 2, insertion cost = 3, deletion cost = 3, length fraction = 0.9, similarity fraction = 0.96. The resulting draft genomes were annotated with the RAST Server 2.0 using the Classic RAST annotation scheme, RAST gene caller, and FIGfams release 70 [25].

**Table 1:** Genome sequences of *V. cholerae* and *V. metoecus* Oyster Pond isolates used in this study

Species and strain	Isolation date	Clonal complex^a^	Genome size (bp)	G+C content (%)	Coverage (×)	N50 (bp)	Completeness (%)^b^	GenBank accession number
*V. cholerae*								
OYP1G01	August, 2009	10	3,969,671	47.4	313	119,133	100	NMTO00000000
OYP2A12	August, 2009	4	4,068,380	47.4	691	174,738	100	NMTN00000000
OYP2E01	August, 2009	2	3,966,741	47.7	292	339,252	100	NMTK00000000
OYP3B05^c^	August, 2009	13	4,014,368	47.5	629	285,424	100	LBGB00000000
OYP3F10	August, 2009	17	3,937,113	47.6	83	94,842	100	NMTJ00000000
OYP4B01	August, 2009	5	3,929,714	47.6	90	128,164	100	NMTI00000000
OYP4C07^c^	August, 2009	13	4,015,430	47.5	714	285,370	100	LBGE00000000
OYP4G08	August, 2009	11	3,927,912	47.5	106	246,196	100	NMTH00000000
OYP4H06	August, 2009	5	3,907,548	47.6	48	133,911	99	NMTG00000000
OYP4H11	August, 2009	2	3,934,959	47.7	154	181,085	100	NMTE00000000
OYP6D06	August, 2009	12	4,036,442	47.6	46	54,501	100	NMTC00000000
OYP6E07	August, 2009	16	3,957,612	47.5	76	145,248	100	NMTB00000000
OYP6F08	August, 2009	2	3,912,172	47.7	134	150,335	100	NMTA00000000
OYP6F10	August, 2009	1	3,860,908	47.6	57	124,026	100	NMSZ00000000
OYP7C09	August, 2009	1	3,869,397	47.6	102	220,009	99	NMSX00000000
OYP8C06	September, 2009	3	4,033,034	47.4	297	210,543	100	NMSV00000000
OYP8F12	September, 2009	3	4,038,901	47.4	366	210,741	100	NMSU00000000
*V. metoecus*								
OP3H^d^	2006	ND^e^	3,963,175	46.9	270	255,052	100	JJMN00000000
OYP4D01^c^	August, 2009	7	3,982,476	46.9	671	253,177	100	LBGO00000000
OYP4E03	August, 2009	1	4,098,312	46.8	302	167,594	100	NMST00000000
OYP5B04^c^	August, 2009	ST1	4,045,661	46.9	719	291,368	100	LBGP00000000
OYP5B06^c^	August, 2009	ND^e^	3,938,456	46.9	802	282,144	100	LBGQ00000000
OYP5H08	August, 2009	7	3,986,064	46.9	386	255,385	100	NMSR00000000
OYP8G05	September, 2009	3	4,040,107	46.9	140	122,729	100	NMSQ00000000
OYP8G09	September, 2009	ND^e^	3,939,012	46.9	171	155,462	100	NMSP00000000
OYP8G12	September, 2009	ST27	3,890,048	46.9	151	254,309	100	NMSO00000000
OYP8H05	September, 2009	6	4,006,704	46.8	171	180,280	100	NMSN00000000
OYP9B03	September, 2009	6	4,016,639	46.8	356	217,439	100	NMSM00000000
OYP9B09	September, 2009	5	4,027,326	46.9	225	136,801	100	NMSL00000000
OYP9C12	September, 2009	ST2	3,924,188	46.9	291	559,905	100	NMSK00000000
OYP9D03^c^	September, 2009	2	3,963,180	46.8	524	277,001	100	LBGR00000000
OYP9D09	September, 2009	1	4,100,806	46.8	379	253,754	100	NMSJ00000000
OYP9E03	September, 2009	2	3,950,427	46.8	171	229,479	100	NMSI00000000
OYP9E10	September, 2009	2	3,914,005	46.8	129	130,891	100	NMSH00000000

^a^Clonal complex and sequence type (ST) information are based on MLSA using seven housekeeping genes by Kirchberger and colleagues [13]. Singletons that do not belong to a clonal complex are indicated by ST numbers instead

^b^Completeness was calculated by determining the presence of 104 core housekeeping genes (Additional file 1)

^c^Also known as YB isolates (YB3B05, YB4C07, YB4D01, YB5B04, YB5B06, YB9D03). Sequenced previously by Orata and colleagues [20]

^d^Sequenced previously by Kirchberger and colleagues [15]

^e^*ND*: not determined

Completeness of genomes was assessed by determining the presence or absence of a subset of core housekeeping genes using BLAST+ 2.5.0 [26]. Completeness in this context was reported as the percentage of 104 single-copy core housekeeping genes present in each genome (Additional file 1). The set of core genes used was modified from Luo and Moran [27] with the addition of genes used for MLSA of the genus *Vibrio* [15, 28–31]. The amino acid sequences of the 104 genes from *V. cholerae* N16961 (GenBank accession nos. NC_002505.1 and NC_002506.1) were used as reference [32]. Putative homologues were decided by calculating the BLAST score ratio (BSR) [33] between reference and query genes and considered if the BSR values were at least 0.3 [34].

### Comparative genomic analyses

The 34 *V. cholerae* and *V. metoecus* genomes were aligned using Mugsy 1.2.3 [35]. Locally collinear blocks less than 500 bp were removed using Galaxy 16.04 [36], and alignment positions with at least one gap were then stripped using Geneious 8.1.2 [37]. The resulting alignment with a total length of 2,801,207 bp was used to reconstruct a maximum-likelihood phylogenetic tree using RAxML 8.0.19 [38] with the general time reversible nucleotide substitution model and gamma model of rate heterogeneity. Robustness of branches was assessed with 100 bootstrap replicates.

Orthologous protein-coding gene families were determined from the annotated genomes using the BPGA tool 1.3.0 [39] with a cutoff of 30% amino acid identity [34]. BPGA was also used to determine the core (100% present in all strains), accessory (present in some strains), and unique (present in one strain only) gene families. Furthermore, species-specific gene families were identified using Intella 1.7.0 [40]. The functions of the gene families were predicted using WebMGA [41] and eggNOG-mapper 2.1.8 [42], based on the Clusters of Orthologous Groups (COG) of proteins database [43]. Gene content was further analyzed using the STRING database 11.5 [44] to elucidate presence or absence of pathways.

To visualize genome comparisons, a BLAST atlas was constructed using GView 1.7 [45] with the *V. cholerae* N16961 genome as reference [32]. The annotated genomes were also scanned for the presence or absence of major genomic islands of *V. cholerae* N16961 [32, 46], as well as known virulence factors of the genus *Vibrio*, available from the Virulence Factor Database (VFDB) [47, 48]. Putative homologues were determined by calculating the BSR [33], as described above.

For every gene family, nucleotide sequences were aligned with ClustalW 2.1 [49]. A maximum-likelihood tree was then reconstructed for each gene alignment using RAxML 8.0.19 [38], as described above. Interspecies gene transfer events were determined and quantified by tree topology comparisons, as described previously [20, 50]. Briefly, the trees were partitioned into clades and visually inspected to determine whether the clades were perfect or not. Following the definition by Schliep and colleagues [50], a perfect clade is a partition that is both complete and homogeneous for a given taxonomic category (e.g., a clade with all *V. cholerae* members and only *V. cholerae*). At least one gene transfer event was hypothesized if a tree showed perfect clades for neither *V. cholerae* nor *V. metoecus* (i.e., in a rooted tree, *V. cholerae* and *V. metoecus* are both polyphyletic). The direction of transfer was then inferred if within a clade of one species (the donor) there was a strain from the other species (the recipient), provided the clustering has robust bootstrap support of at least 70% [51]. For example, a gene transfer from *V. cholerae* to *V. metoecus* is inferred if a strain of *V. metoecus* clusters within the *V. cholerae* clade in that gene tree.

### Statistical analysis of HGT events

Statistical analyses of total HGT events between *V. cholerae* and *V. metoecus* were assessed separately for the core and accessory gene datasets using QI Macros 2018 [52]. An Anderson–Darling test for normality was performed for each grouping. Accordingly, the accessory gene dataset used *t*-test assuming equivalent variances, while the non-parametric Mann–Whitney–Wilcoxon test was used on the core gene dataset. Differences were acknowledged as statistically significant at *p*-values < 0.05.

### Quality assurance

To obtain a pure culture, each strain was repeatedly subcultured (three times) on TSA while ensuring appropriate aseptic techniques. A single colony was then inoculated into TSB for overnight culturing and for use in genomic DNA extraction. Only high-quality genomes that were complete (100%) or near complete (99%) were used in this study (Table 1) based on our completeness assessment (see above). After preliminary identification of strains by MLSA (see above), species identities of the sequenced genomes were verified to be *V. cholerae* or *V. metoecus* by determining the average nucleotide identity (ANI) against known reference/type strain genomes, *V. cholerae* N16961 or *V. metoecus* OP3H (JJMN00000000), and against each other. Comparisons among *V. cholerae* genomes exhibited 98–100% ANI. Comparisons among *V. metoecus* genomes exhibited 97–100% ANI. These are well above the 95% ANI cutoff for genomes to belong to the same species [53]. On the other hand, comparisons between *V. cholerae* and *V. metoecus* genomes resulted in 86–87% ANI only.

## Results and discussion

### Genes exclusively present in *V. cholerae* or *V. metoecus* are potential phenotypic markers for species identification

**Fig. 1:**
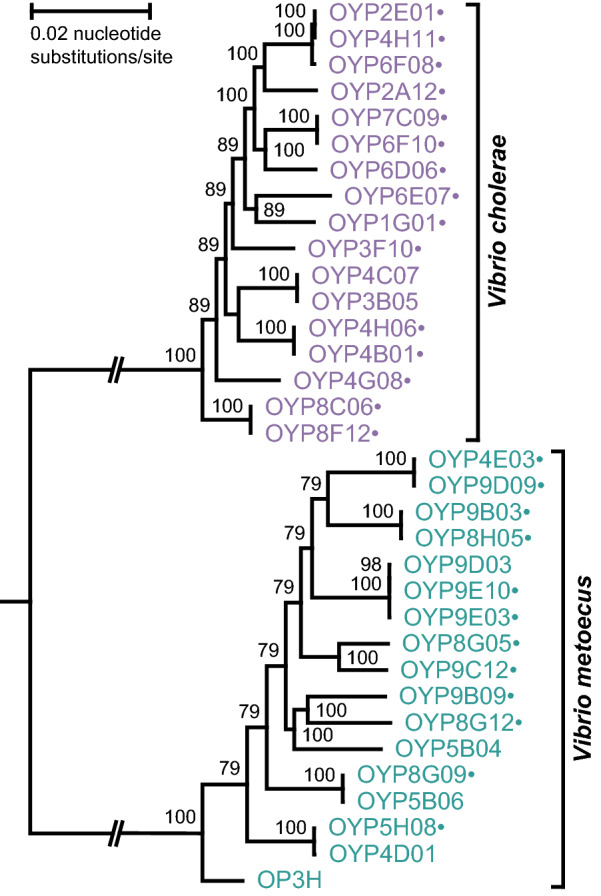
The phylogenetic relationship of *V. cholerae* and *V. metoecus*. The phylogeny was reconstructed from a core-genome alignment of ~ 2.8 Mb in length. Bootstrap values are indicated on the nodes. The scale bar represents 0.02 nucleotide substitutions per site. Parallel lines indicate shortened branch lengths, approximately 5× the scale bar. Dots after the strain names indicate genomes sequenced in this study

To obtain a clearer picture of genetic differences between *V. cholerae* and *V. metoecus*, we sequenced genomes of strains from both species co-isolated from Oyster Pond, a cholera-free environment in the US East Coast [13, 19]. The rarity of *V. metoecus* limited the size of the dataset, because only 17 *V. metoecus* isolates with high quality genome sequences could be obtained (5 from previous studies [15, 20] and 12 in this study). An equal number of *V. cholerae* genomes of strains isolated from the same location were therefore selected (2 from a previous study [20] and 15 from this study), matching the genetic diversity of the *V. metoecus* genomes based on clonal complex or sequence type previously determined by MLSA [13] to avoid bias (Table 1). A whole-genome-based phylogenetic tree clearly clustered our 17 *V. cholerae* and 17 *V. metoecus* isolates in two distinct clades with robust bootstrap support (Fig. 1).

**Fig. 2:**
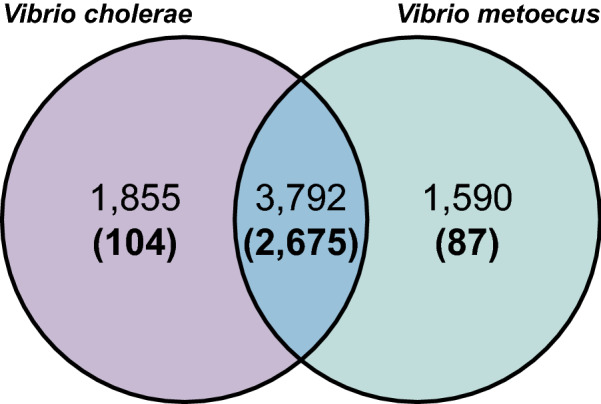
Distribution of core genes and species-specific genes between *V. cholerae* and *V. metoecus*. The Venn diagram shows the number of genes shared between *V. cholerae* and *V. metoecus* or present within each species. Top numbers indicate total gene families in the pan-genome of both species. Bottom numbers (in bold and parentheses) indicate gene families exclusively present in all strains of *V. cholerae*, *V. metoecus*, or both

Subsequently, the pan-genome of the two species was categorized into either core or species-specific gene families (Fig. 2). With this larger dataset, metabolic differences between the species could be found, such as the ability of *V. metoecus* to use D-glucuronic acid and N-acetyl-D-galactosamine as sole carbon sources [15] (COG3250)﻿. These glycosaminoglycans are present in the extracellular matrix or attached to the membrane of eukaryotic cells. Pathogenic bacteria express various adhesins to bind to these structures prior to colonization and infection, as in pulmonary infections by *Bordetella pertussis* and *Mycobacterium tuberculosis* and urogenital infections by *Chlamydia trachomatis* and *Neisseria gonorrhoeae* [54].

Another striking difference between the species is in nitrogen metabolism. Although both species display pathways for nitrate reduction, only *V. metoecus* harbors enzymes used for nitrite reduction to ammonia (COG3301, COG3303) (Additional file 2). Nitrite reductases have also been implicated in virulence [55]. For example, pathogens such as *M. tuberculosis*, *N. gonorrhoeae*, and *Pseudomonas aeruginosa* reduce nitrite—a byproduct produced by mammals in low oxygen conditions such as in the gut—to less toxic secondary components like nitrous oxide or ammonia using nitrite reductases, giving them selective advantage to survive in the harsh environment in the host [55, 56]. This suggests that *V. metoecus* could also have an advantage growing under anaerobic conditions if nitrite is available [57, 58]. For its part, *V. cholerae* has a multisubunit Na^+^/H^+^ antiporter complex (COG1006, COG1863, COG2212, COG1320) involved in salt and pH tolerance, which could have a significant effect on its niche (Additional file 3). *V. cholerae* also harbors a superoxide dismutase (COG0605) that is absent in *V. metoecus*, which could aid with resistance to phagocytosis by predatory protists or phagocytes [59].

Two other genes unique to *V. metoecus* potentially suited to phenotypic assays to differentiate it from *V. cholerae* were identified (Additional file 2). The first is a gene encoding a rhodanese-related sulfurtransferase (COG2897). Rhodanese is widely distributed in *Bacteria*, *Archaea*, and *Eukarya*, and studied mainly for its role in sulfur metabolism and cyanide detoxification [60–62]. The direct role of bacterial rhodanese in pathogenicity, if any, is yet to be investigated. Meanwhile, studies on other model systems show that rhodanese from a ruminant nematode parasite (*Haemonchus contortus*) can bind to peripheral blood mononuclear cells of its mammalian host (goat) and results in a poor host immune response [63]. This finding is interesting since a clinical strain of *V. metoecus* was extracted from blood [20], indicating that strains of this species can be opportunistic pathogens, where rhodanese may also play a role in its pathogenicity. A test for rhodanese activity in bacteria for phenotypic differentiation using the EDTA-lysozyme cell lysis method has been developed previously [62, 64].

The second gene encodes peptidase E (PepE) (COG3340), an enzyme involved in the degradation of proteins to free amino acids [65]. In addition to direct nutrient acquisition, peptidases also play a role in pathogenicity. During invasion of host cells and tissues, gut pathogens can secrete peptidases to degrade host proteins and competitively colonize the gut [66]. However, unlike many peptidases with broad substrate specificities, PepE is unique as it has a very specific target (N-terminal aspartic dipeptides). Interestingly, PepE is not as widespread as the broad-specificity peptidases. A survey of microbial genomes for PepE homologues revealed that its distribution is mainly limited to *Gammaproteobacteria* (*Escherichia coli*, *Haemophilus influenzae*, *Salmonella enterica* sv. Typhimurium, among others) [65]. *V. metoecus* PepE has the closest homologs to those found in other distant vibrios (*Vibrio alginolyticus*, *Vibrio parahaemolyticus*, *Vibrio vulnificus*, among others). Among the more closely related members of the Cholerae clade, which includes both *V. metoecus* and *V. cholerae* [16], PepE is only found in one other species (*Vibrio mimicus*). The fact that PepE is not so widespread suggests that it could serve a very specialized and possibly advantageous function to organisms that encode it [65]. PepE activity can be assessed using a previously established assay [67] that can be modified specifically for vibrios.

### Pathogenic potential of *V. cholerae* and *V. metoecus* from Oyster Pond

The two major virulence factors of toxigenic *V. cholerae* are the cholera toxin (CTX) and toxin-coregulated pilus (TCP), both acquired horizontally by phage infections [68, 69]. The cholera toxin, encoded by the *ctxAB* genes, is a potent enterotoxin that is responsible for the profuse watery diarrhea in cholera patients [68]. The TCP, encoded by genes of the TCP cluster, is a crucial factor to establish colonization in the host intestine [70] and serves as a receptor for the CTX phage [69]. These virulence factors are not present in any of the Oyster Pond isolates (Additional file 4), suggesting that these environmental isolates cannot produce the cholera toxin and are not capable of acquiring the genomic island that encodes it.

**Fig. 3:**
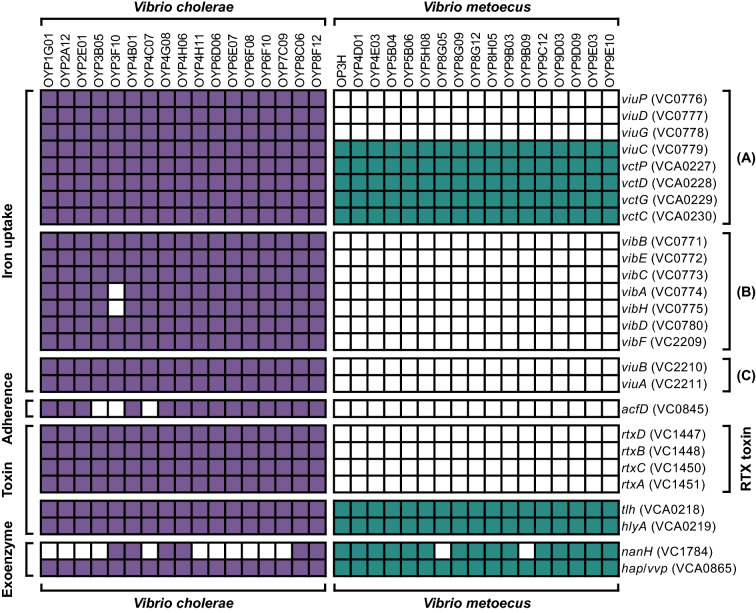
Presence/absence map of selected virulence factors in *V. cholerae* and *V. metoecus*. The table of virulence factors for the genus *Vibrio* was obtained and modified from the VFDB [47, 48]. Colored squares represent BSRs of at least 0.3 against reference genes (i.e., homologues of genes are present); white squares represent absence of genes. The genes involved in iron uptake are further categorized into (**A**) periplasmic binding protein-dependent ABC transport systems, (**B**) vibriobactin biosynthesis, and (**C**) vibriobactin utilization. Locus tags of the genes in the *V. cholerae* N16961 reference genome are indicated after the gene names

Nonetheless, both *V. cholerae* and *V. metoecus* encode several virulence factors such as metalloprotease (*hap/vvp*), thermolabile hemolysin (*tlh*), and cytolysin (*hlyA*), implicated in extra-intestinal infections (Fig. 3). For example, metalloproteases have been demonstrated in *Vibrio* species to degrade biologically relevant host proteins such as collagen, mucin, and gelatin [71]. Hemolysin and cytolysin are able to permeabilize cell membranes through the formation of pores and induce apoptosis in various animal cell models [72]. Iron bioavailability is strictly regulated within the host as part of an innate immune response during an infection; hemolysins can lyse red blood cells with iron-chelating proteins to supersede the restriction [72, 73]. They also encode the type VI secretion system (T6SS) for the contact-dependent killing of nearby cells (Additional file 4) [74]. The T6SS in *V. cholerae* and *V. metoecu*s Oyster Pond isolates possesses a diverse and complex arrangement of effector toxins that can be lethal to bacteria and/or eukaryotes [75, 76], although it has been demonstrated previously that *V. metoecus* is less effective at defending against eukaryotic predation [15]. *V. metoecus* RC341, an environmental isolate from Chesapeake Bay, Maryland, USA, contains similar virulence factors in its genome [18]. Additionally, both *V. metoecus* and *V. cholerae* have evidence of species-specific multidrug efflux pumps (Additional files 2 and 3, respectively), indicating the potential for antibiotic resistance.

Multiple virulence factors are present only in the 17 *V. cholerae* isolates but not in *V. metoecus*, including genes involved in iron uptake, colonization, and toxin production (Fig. 3). Iron is an essential nutrient but is a very limited resource in the host, and this has shaped the evolution of survival strategies and bactericidal defense mechanisms of pathogens [77]. The presence of various iron uptake genes in the environmental strains of *V. cholerae* but not *V. metoecus* could facilitate survival in the intestine after exposure by ingestion. In addition, 14 *V. cholerae* isolates have the *acfD* gene, part of a cluster of four genes (*acfABCD*) called the accessory colonization factor also involved in the colonization of the intestine but distinct from the TCP cluster [78]. Gene *acfD* was not found in other non-Oyster Pond *V. metoecus* strains, including clinical isolates [20]. However, none of the *V. cholerae* nor *V. metoecus* Oyster Pond isolates have the *acfABC* genes (Additional file 4), whereas known clinical and non-Oyster Pond environmental strains of *V. cholerae* have the complete cluster [20]. Lastly, the RTX gene cluster is only present in *V. cholerae* and absent in *V. metoecus*. In this cluster, RtxA causes the rounding and death of eukaryotic cells by catalyzing covalent cross-linking of cellular actin through its actin-crosslinking domain [79]. This gene cluster could provide a more robust anti-eukaryotic response to *V. cholerae*, which has been shown to survive predation by slime molds better than *V. metoecus* [15].

On the other hand, neuraminidase (*nanH*) is present in 15 *V. metoecus* isolates, but only present in 6 *V. cholerae* (Fig. 3). In toxin-producing organisms, neuraminidase cleaves sialic acid of gangliosides on host cell surfaces, exposing the inner core sugars that then serves as receptors for the toxin [80]. In vibrios, the neuraminidase cluster is part of the *Vibrio* pathogenicity island – 2, a major genomic island initially identified only in toxigenic *V. cholerae* [81]. However, fragments of this island have been detected in environmental *V. cholerae*, as well as in environmental and clinical *V. metoecus* [20]. Specifically, an almost complete neuraminidase cluster is present in all but one *V. metoecus* isolates from this study (Additional file 5). Since these isolates are not toxigenic (i.e., no *ctxAB* genes), neuraminidase is likely used for nutrient acquisition [82].

### HGT inference suggests a directional bias in gene transfer from *V. cholerae* to *V. metoecus*

**Fig. 4:**
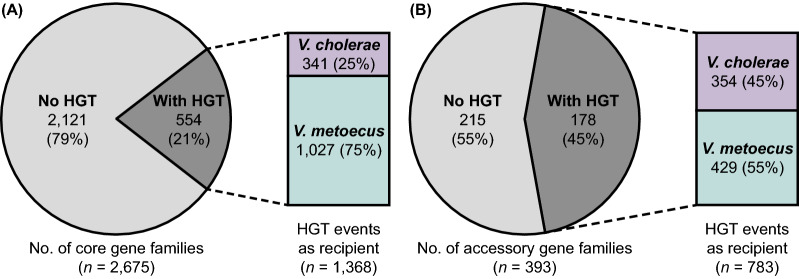
Inferred HGT events in the core and accessory genomes. The pie charts show the number of (**A**) core and (**B**) accessory gene families with and without HGT. The bars show the number of HGT events within the gene families, showing the frequency of *V. cholerae* or *V. metoecus* as recipients

It has previously been suggested that *V. cholerae* and *V. metoecus* frequently exchange genes through HGT, although with a limited dataset sourced from various geographical locations and a mixture of clinical and environmental strains [19, 20]. To verify this hypothesis, the core genome of the two species was determined for our genome dataset (Fig. 2). A maximum-likelihood tree was reconstructed for each of the 2675 core gene families. Phylogenetic trees were also reconstructed for the accessory genes. We only examined accessory gene families present in at least 17 genomes (half of the number genomes, 621 gene families in total) (Additional file 6). A gene transfer event and its directionality were inferred in a tree, whether from *V. cholerae* to *V. metoecus* or vice versa (see Methods for details on HGT inference). Out of the 2675 core gene family trees, 554 (20.7%) could not be partitioned into perfect clades (i.e., has at least one HGT event) (Fig. 4A and Additional file 7). From the 554 trees exhibiting HGT, 1368 gene transfer events were inferred, where *V. metoecus* was a recipient of genes from *V. cholerae* in 1027 (75.1%) of those transfer events, whereas *V. cholerae* was a recipient in only 341 (24.9%). Additionally, from the 621 trees of accessory gene families, 228 were excluded from our analysis, as they consisted of members from one species only or did not have robust bootstrap support (< 70%) to confidently infer HGT. From the remaining 393 trees, 178 (45.3%) exhibited interspecies HGT, more than twice the proportion (21%) found in the core genome (Fig. 4B and Additional file 8). Among the gene families from the accessory genome that could be analyzed, *V. metoecus* was a recipient in 429 out of 783 (54.8%) transfer events, while *V. cholerae* was a recipient in 354 (45.2%). Considering both core and accessory genomes, there is a higher number of gene transfer events from *V. cholerae* to *V. metoecus*, indicating a bias in the direction of gene transfer. This is more prominent within the core genome, where *V. metoecus* was three times more often the recipient of genes from *V. cholerae* (a statistically significant difference, *p* < 0.001) (Additional file 7)*.* In contrast, HGT directionality was only 1.2× higher in favour of *V. metoecus* as the recipient in the accessory genome (not statistically significant, *p* = 0.114) (Additional file 8). The accessory genes might encode supplementary functions that are not necessarily essential for growth but may offer selective advantages, such as niche adaptation, antibiotic resistance, or host colonization [83]. On the contrary, the core genome, which contains genes essential for growth, is expected to be under strong selective pressure, limiting the extent of sequence changes and preventing gene loss [84], which could explain the lesser frequency of transfers in the core genes than accessory genes. However, the more pronounced HGT directional bias observed for the core genes as opposed to accessory genes could be linked to the fact that the core genes are more abundant since they are present in all members of the population [83].

A possible explanation for the observed bias in HGT towards *V. metoecus* is that *V. cholerae* could be more refractory to gene acquisition, containing more barriers to gene uptake or less efficient DNA uptake systems. Thus, we surveyed our annotated genomes for the presence or absence of genes that are involved in the regulation of competence or provide barriers to DNA uptake. Major regulators and genes of the DNA uptake system [85–87] are present in all of our isolates (Additional file 9). On the other hand, some isolates of *V. cholerae* and *V. metoecus* were missing some or all genes of the restriction-modification system, an immune system in bacteria that recognize self from non-self (foreign) DNA [88]. Nuclease activity can also inhibit natural transformation [89, 90], and the genes encoding the deoxyribonucleases Dns and Xds are present in all our isolates of both species. There is therefore no clear difference in genotype that could explain the HGT bias observed.

It is hypothesized that one of the reasons for the bias in HGT directionality could be due to a higher abundance of *V. cholerae* than *V. metoecus* in the environment [20]. In Oyster Pond, *V. cholerae* is three times more abundant than *V. metoecus* on average when both species are present [23]. This higher abundance would lead to having more DNA from *V. cholerae* readily available for acquisition by *V. metoecus*, making it a decisive factor in biasing HGT directionality. Additionally, seasonal abundance patterns have been found for *V. cholerae*, which is more abundant in the warmer months of the year (May to September) and rarely detectable in winter [91]. Thus, it is also hypothesized that seasonality could play a role in this bias. The seasonal abundance of *V. metoecus* was determined previously and is much more striking than *V. cholerae*. The former species is essentially limited to the last 2 months of summer in Oyster Pond (August and September) [23]. This bloom is remarkable, as *V. metoecus* would need to compete with established *V. cholerae* and several other species in the same environment. It is possible that strong competition from blooming *V. metoecus* would release *V. cholerae* genetic material upon lysis, promoting integration of new genetic material in surviving competitors. The more pronounced bottlenecks in the *V. metoecus* population could also lead to many genes acquired from *V. cholerae* during the blooms being lost from the population (and therefore resulting in the directional bias observed) [92]. It is unclear if the abundance and seasonality differences observed between *V. cholerae* and *V. metoecus* in the Oyster Pond ecosystem is also present in other environments. Intensive molecular and culture-based surveys of coastal and inland water regions of Bangladesh have failed to find any *V. metoecus* so far despite detecting abundant *V. cholerae* [23], suggesting that the two organisms have a different geographical distribution and that the latter might be far more ubiquitous.

## Conclusion

Comparison of the gene repertoires of a larger number of *V. metoecus* genomes obtained in this study sheds further insight into the biology of the poorly understood species, a close relative of *V. cholerae*. Additional genes unique to *V. metoecus* were identified—for nitrite reduction, rhodanese-related sulfurtransferase, and peptidase E—which could potentially be used in phenotypic tests to differentiate it from *V. cholerae*, as well as from more distant vibrios. Additionally, since the discovery of *V. metoecus* [15], two more very close relatives of *V. cholerae* have been described (*Vibrio paracholerae* [93] and *Vibrio tarriae* [16]), both of which have also been associated with human infections. As more strains are being identified as potentially novel sister species to *V. cholerae* [16, 93–96], our understanding of these species is also evolving. Therefore, an update on the diagnostic tests for *V. cholerae* (and close relatives) is needed to incorporate new knowledge from recently discovered sister species, which could easily be misdiagnosed as *V. cholerae*.

Furthermore, the co-isolation of *V. metoecus* and *V. cholerae* has provided the opportunity to study the extent of HGT between closely related species in a natural environment. Here, the correlation between the rate of HGT and the abundance of donor and recipient species is suggested. Although the direct link between abundance and rate of HGT has not been experimentally demonstrated, there is an exact correlation between these two factors in a natural environment [23], therefore warranting further investigation. It should be noted that the majority of bacteria in nature (up to 80%) are estimated to exist in biofilms [97], which form multispecies communities that facilitate resource and gene exchange, competition, cell signaling, and resistance to environmental stressors such as desiccation [98–101]. Within these complex assemblies, and in environmental reservoirs in general, HGT is expected to occur outside of the instances we have suggested between *V. cholerae* and *V. metoecus*, which may have contributed to the gene acquisitions we have observed. Interestingly, we find potential instances of interspecific HGT in examples such as *V. metoecus* possessing nitrite reductase genes. As denitrification occurs in many aquatic environmental reservoirs, it can be posited that the acquisition of these genes may have occurred with a donor outside of *V. cholerae* or *V. metoecus*. However, this would need to be assessed with metagenomics analyses, which is outside of the scope of this work. Nonetheless, the majority of HGT has been demonstrated to happen intraspecifically or between closely related species [102–104]. As both *Vibrio* species are closely related, and a careful genetic screen also shows that there is no reason to suspect a difference in ability between the two species to uptake DNA, we hypothesize that differential abundance is the most likely explanation for the HGT bias reported in this study. Observation of this correlation in various geographic locations would strengthen this hypothesis. If a direct relationship between relative abundance of a species in a community and its intake of foreign DNA is confirmed for these vibrios and other bacterial species, it would have major implications for the dynamics of speciation and the spread of various fitness characteristics between species.

## Supplementary Information

**Additional file 1**: Core housekeeping genes used to determine genome completeness.

**Additional file 2**: Predicted functions of genes found in *V. metoecus* but not in *V. cholerae*.

**Additional file 3**: Predicted functions of genes found in *V. cholerae* but not in *V. metoecus*.

**Additional file 4**: Presence/absence map of virulence factors in *V. cholerae* and *V. metoecus*.

**Additional file 5**: Presence/absence map of genes of the *Vibrio *pathogenicity island – 2 in *V. cholerae* and *V. metoecus.*

**Additional file 6**: The pan-genome of *V. cholerae* and *V. metoecus*.

**Additional file 7**: Count of horizontal gene transfer events within the core genes of *V. cholerae* and *V. metoecus*.

**Additional file 8**: Count of horizontal gene transfer events within the accessory genes of *V. cholerae* and *V. metoecus*.

**Additional file 9**: Presence/absence map of genes involved in DNA uptake and restriction-modification, as well as endo- and exonucleases in *V. cholerae* and *V. metoecus*.

## Data Availability

The genome datasets supporting the conclusions of this article are available in the GenBank repository under BioProject accession number PRJNA281423. The individual GenBank accession numbers are listed in Table 1.

## Abbreviations

ANI: Average nucleotide identity
BSR: BLAST score ratio
COG: Clusters of Orthologous Groups
CTX: Cholera toxin
HGT: Horizontal gene transfer
MLSA: Multilocus sequence analysis
ST: Sequence type
TCBS: Thiosulfate citrate bile sucrose
TCP: Toxin-coregulated pilus
TSA: Tryptic soy agar
TSB: Tryptic soy broth
T6SS: Type VI secretion system
VFDB: Virulence Factor Database
