# Supplementary material for: Genomes of Vibrio metoecus co-isolated with Vibrio cholerae extend our understanding of differences between these closely related species
Source: Gut Pathog. 2022 Nov 20;14:42. doi: 10.1186/s13099-022-00516-x (PMC9677704; doi:10.1186/s13099-022-00516-x)
Supplement: Supplementary file 1 — Additional file 1: Core housekeeping genes used to determine genome completeness. [file 13099_2022_516_MOESM1_ESM.pdf]

Additional file 1. Core housekeeping genes used to determine genome completeness

Gene	Gene description	Accession no.[a]
adk	Adenylate kinase	NP_230632
alas	Alanyl-tRNA synthetase	NP_230196
argS	Arginyl-tRNA synthetase	NP_231706
aroA	5-Enolpyruvylshikimate-3-phosphate synthetase	NP_231368
aroC	Chorismate synthase	NP_231747
aspS	Aspartyl-tRNA synthetase	NP_230811
birA	Biotin-[acetylCoA carboxylase] holoenzyme synthetase	NP_229973
coaE	Dephospho-CoA kinase	NP_232057
cysS	Cysteinyl-tRNA synthetase	NP_231482
dfp	Fused 4'-phosphopantothenolcysteine decarboxylase	NP_229872
dnaE	DNA polymerase III, alpha subunit	NP_231876
dnaN	DNA polymerase III, beta subunit	NP_062597
efp	Elongation factor EF-P	NP_230854
eno	Enolase	NP_232076
ffh	Signal recognition particle (SRP) component with 4.5S RNA	NP_230211
folP	7,8-Dihydropteroate synthase	NP_230287
frr	Ribosome recycling factor	NP_231888
ftsY	Fused signal recognition particle (SRP) receptor	NP_229805
fusA	Protein chain elongation factor EF-G, GTP-binding	NP_230015
gyrB	DNA gyrase subunit B	NP_229675
ileS	Isoleucyl-tRNA synthetase	NP_230331
infB	Fused protein chain initiation factor 2, IF-2	NP_230292
infC	Protein chain initiation factor, IF-3	NP_232684
lepA	GTP-binding membrane protein	NP_232092
leuS	Leucyl-tRNA synthetase	NP_230603
mdh	Malate dehydrogenase	NP_230086
metG	Methionyl-tRNA synthetase	NP_230681
ndk	Nucleoside diphosphate kinase	NP_230405
nusB	Transcription antitermination protein	NP_231898
pgi	Glucose-6-phosphate isomerase	NP_230028
pheS	Phenylalanine tRNA synthetase, alpha subunit	NP_230864
pheT	Phenylalanine tRNA synthetase, beta subunit	NP_230865
pncB	Nicotinate phosphoribosyltransferase	NP_232499
pnp	Polynucleotide phosphorylase/polyadenylase	NP_230296
prfA	Peptide chain release factor RF-1	NP_231810
priA	Primosome factor n' (replication factor Y)	NP_232306
pros	Prolyl-tRNA hydrolase	NP_230522
pstA	Phosphate transporter subunit	NP_232473
pth	Peptidyl-tRNA hydrolase	NP_231815
purA	Adenylosuccinate synthetase	NP_232230
pyrB	Aspartate carbamoyltransferase, catalytic subunit	NP_232139
pyrF	Orotidine-5'-phosphate decarboxylase	NP_231545
pyrG	CTP synthetase	NP_232077
pyrH	Uridylate kinase	NP_231889
recA	DNA strand exchange and recombination protein	NP_230194
recG	ATP-dependent DNA helicase	NP_232338
purC	Phosphoribosylaminoimidazole-succinocarboxamide synthetase	NP_230835
rnc	RNase III	NP_232090
rnhB	Ribonuclease HII	NP_231877
rplA	50S ribosomal subunit protein L1	NP_229979
rplB	50S ribosomal subunit protein L2	NP_232221
rplC	50S ribosomal subunit protein L3	NP_232224
rplD	50S ribosomal subunit protein L4	NP_232223
rplE	50S ribosomal subunit protein L5	NP_232212

Gene	Gene description	Accession no.[a]
rplE	50S ribosomal subunit protein L5	NP_232212
rplF	50S ribosomal subunit protein L6	NP_232209
rplK	50S ribosomal subunit protein L11	NP_229978
rplL	50S ribosomal subunit protein L7/12	NP_229981
rplM	50S ribosomal subunit protein L13	NP_230221
rplN	50S ribosomal subunit protein L14	NP_232214
rplO	50S ribosomal subunit protein L15	NP_232205
rplP	50S ribosomal subunit protein L16	NP_232217
rplQ	50S ribosomal subunit protein L17	NP_232198
rplR	50S ribosomal subunit protein L18	NP_232208
rplS	50S ribosomal subunit protein L19	NP_230215
rplT	50S ribosomal subunit protein L20	NP_232686
rplV	50S ribosomal subunit protein L22	NP_232219
rplX	50S ribosomal subunit protein L24	NP_232213
rpoA	RNA polymerase, alpha subunit	NP_232199
rpoB	RNA polymerase, beta subunit	NP_229982
rpsB	30S ribosomal subunit protein S2	NP_231891
rpsC	30S ribosomal subunit protein S3	NP_232218
rpsD	30S ribosomal subunit protein S4	NP_232200
rpsE	30S ribosomal subunit protein S5	NP_232207
rpsG	30S ribosomal subunit protein S7	NP_230014
rpsH	30S ribosomal subunit protein S8	NP_232210
rpsI	30S ribosomal subunit protein S9	NP_230222
rpsJ	30S ribosomal subunit protein S10	NP_232225
rpsK	30S ribosomal subunit protein S11	NP_232201
rpsL	30S ribosomal subunit protein S12	NP_230013
rpsM	30S ribosomal subunit protein S13	NP_232202
ruvA	Component of RuvABC resolvasome, regulatory subunit	NP_231480
ruvB	DNA helicase, component of RuvABC resolvasome	NP_231479
secA	Preprotein translocase subunit, ATPase	NP_232024
secY	Preprotein translocase membrane subunit	NP_232204
serS	Seryl-tRNA synthetase	NP_230755
smpB	Trans-translation protein	NP_230495
tgt	tRNA-guanine transglycosylase	NP_230390
thrS	Threonyl-tRNA synthetase	NP_232683
tig	Peptidyl-prolyl cis/trans isomerase A	NP_231930
toxR	Cholera toxin transcriptional activator	NP_230630
tpiA	Triosephophate isomerase	NP_232298
trmD	tRNA (guanine-1-) -methyltransferase	NP_230214
trpA	Tryptophan synthase, alpha subunit	NP_230814
trpC	Indole-3-glycerol phosphate synthase	NP_230816
trpS	Tryptophanyl-tRNA synthetase	NP_232251
truB	tRNA pseudouridine synthase B	NP_230294
tsf	Protein chain elongation factor EF-Ts	NP_231890
tyrS	Tyrosyl-tRNA synthetase	NP_230119
uvrA	ATPase and DNA damage recognition protein	NP_230048
uvrB	Exonuclease of nucleotide excision repair	NP_230664
valS	Valyl-tRNA synthetase	NP_232132
yabC	S-Adenosyl-dependent methyltransferase activity	NP_232039
ybeY	Conserved protein	NP_230607
yidC	Cytoplasmic insertase, Sec system	NP_062588

[a] Accession numbers are from the reference genome *V. cholerae* N16961
