# Supplementary material for: Genomes of Vibrio metoecus co-isolated with Vibrio cholerae extend our understanding of differences between these closely related species
Source: Gut Pathog. 2022 Nov 20;14:42. doi: 10.1186/s13099-022-00516-x (PMC9677704; doi:10.1186/s13099-022-00516-x)
Supplement: Supplementary file 2 — Additional file 2: Predicted functions of genes found in V. metoecus but not in V. cholerae. [file 13099_2022_516_MOESM2_ESM.pdf]

Additional file 2. Predicted functions[a] of genes found in *V. metoecus*[b] but not in *V. cholerae*

COG hit	Description	Class/es	Class description/s
COG0004	Ammonia permease	P	Inorganic ion transport and metabolism
COG0247	Fe-S oxidoreductase	C	Energy production and conversion
COG0346	Lactoylglutathione lyase and related lyases	E	Amino acid transport and metabolism
COG0437	Fe-S-cluster-containing hydrogenase components 1	C	Energy production and conversion
COG0471	Di- and tricarboxylate transporters	P	Inorganic ion transport and metabolism
COG0493	NADPH-dependent glutamate synthase beta chain and related oxidoreductases	E	Amino acid transport and metabolism
COG0526	Thiol-disulfide isomerase and thioredoxins	OC	Posttranslational modification, protein turnover, and chaperones; energy production and conversion
COG0534	Na+-driven multidrug efflux pump	V	Defense mechanisms
COG0614	ABC-type Fe3+-hydroxamate transport system, periplasmic component	P	Inorganic ion transport and metabolism
COG0671	Membrane-associated phospholipid phosphatase	I	Lipid transport and metabolism
COG0739	Membrane proteins related to metalloendopeptidases	M	Cell wall/membrane/envelope biogenesis
COG1018	Flavodoxin reductases (ferredoxin-NADPH reductases) family 1	C	Energy production and conversion
COG1138	Cytochrome c biogenesis factor	O	Posttranslational modification, protein turnover, and chaperones
COG1151	6Fe-6S prismane cluster-containing protein	C	Energy production and conversion
COG1177	ABC-type spermidine/putrescine transport system, permease component II	E	Amino acid transport and metabolism
COG1252	NADH dehydrogenase, FAD-containing subunit	C	Energy production and conversion
COG1263	Phosphotransferase system IIC components, glucose/maltose/N-acetylglucosamine-specific	G	Carbohydrate transport and metabolism
COG1664	Integral membrane protein CcmA involved in cell shape determination	M	Cell wall/membrane/envelope biogenesis
COG1670	Acetyltransferases, including N-acetylases of ribosomal proteins	J	Translation, ribosomal structure, and biogenesis
COG1840	ABC-type Fe3+ transport system, periplasmic component	P	Inorganic ion transport and metabolism
COG1914	Mn2+ and Fe2+ transporters of the NRAMP family	P	Inorganic ion transport and metabolism
COG2188	Transcriptional regulators	K	Transcription
COG2195	Di- and tripeptidases	E	Amino acid transport and metabolism
COG2897	Rhodanese-related sulfurtransferase	P	Inorganic ion transport and metabolism
COG2931	RTX toxins and related Ca2+-binding proteins	Q	Secondary metabolites biosynthesis, transport, and catabolism
COG3061	Cell envelope opacity-associated protein A	M	Cell wall/membrane/envelope biogenesis
COG3069	C4-dicarboxylate transporter	C	Energy production and conversion
COG3088	Uncharacterized protein involved in biosynthesis of c-type cytochromes	O	Posttranslational modification, protein turnover, and chaperones
COG3170	Tfp pilus assembly protein FimV	NU	Cell motility; intracellular trafficking, secretion, and vesicular transport
COG3250	Beta-galactosidase/beta-glucuronidase	G	Carbohydrate transport and metabolism
COG3301	Formate-dependent nitrite reductase, membrane component	P	Inorganic ion transport and metabolism
COG3303	Formate-dependent nitrite reductase, periplasmic cytochrome c552 subunit	P	Inorganic ion transport and metabolism
COG3340	Peptidase E	E	Amino acid transport and metabolism
COG3486	Lysine/ornithine N-monooxygenase	Q	Secondary metabolites biosynthesis, transport, and catabolism
COG3710	DNA-binding winged-HTH domains	K	Transcription
COG3842	ABC-type spermidine/putrescine transport systems, ATPase components	E	Amino acid transport and metabolism
COG4191	Signal transduction histidine kinase regulating C4-dicarboxylate transport system	T	Signal transduction mechanisms
COG4235	Cytochrome c biogenesis factor	O	Posttranslational modification, protein turnover, and chaperones
COG4566	Response regulator	T	Signal transduction mechanisms
COG5001	Predicted signal transduction protein containing a membrane domain, an EAL and a GGDEF domain	T	Signal transduction mechanisms

[a] Functions are categorized based on the Clusters of Orthologous Groups (COG) of proteins database

[b] There are a total of 87 putative unique genes found in *V. metoecus*. Not included in this table are genes that have general function predictions only (n = 28), encode hypothetical proteins (n = 7), or have no known hits (n = 12)
