# Supplementary material for: Genomes of Vibrio metoecus co-isolated with Vibrio cholerae extend our understanding of differences between these closely related species
Source: Gut Pathog. 2022 Nov 20;14:42. doi: 10.1186/s13099-022-00516-x (PMC9677704; doi:10.1186/s13099-022-00516-x)
Supplement: Supplementary file 3 — Additional file 3: Predicted functions of genes found in V. cholerae but not in V. metoecus. [file 13099_2022_516_MOESM3_ESM.pdf]

Additional file 3. Predicted functions[a] of genes found in *V. cholerae*[b] but not in *V. metoecus*

COG hit	Description	Class/es	Class description/s
COG0028	Thiamine pyrophosphate-requiring enzymes [acetolactate synthase, pyruvate dehydrogenase (cytochrome), glyoxylate carboligase, phosphonopyruvate decarboxylase]	EH	Amino acid transport and metabolism; coenzyme transport and metabolism
COG0242	N-formylmethionyl-tRNA deformylase	J	Translation, ribosomal structure, and biogenesis
COG0574	Phosphoenolpyruvate synthase/pyruvate phosphate dikinase	G	Carbohydrate transport and metabolism
COG0589	Universal stress protein UspA and related nucleotide-binding proteins	T	Signal transduction mechanisms
COG0601	ABC-type dipeptide/oligopeptide/nickel transport systems, permease components	EP	Amino acid transport and metabolism; inorganic ion transport and metabolism
COG0605	Superoxide dismutase	P	Inorganic ion transport and metabolism
COG0651	Formate hydrogenlyase subunit 3/Multisubunit Na+/H+ antiporter, MnhD subunit	CP	Energy production and conversion; inorganic ion transport and metabolism
COG0654	2-polyprenyl-6-methoxyphenol hydroxylase and related FAD-dependent oxidoreductases	HC	Coenzyme transport and metabolism; energy production and conversion
COG0664	cAMP-binding proteins - catabolite gene activator and regulatory subunit of cAMP-dependent protein kinases	T	Signal transduction mechanisms
COG0745	Response regulators consisting of a CheY-like receiver domain and a winged-helix DNA-binding domain	TK	Signal transduction mechanisms; transcription
COG0747	ABC-type dipeptide transport system, periplasmic component	E	Amino acid transport and metabolism
COG0789	Predicted transcriptional regulators	K	Transcription
COG0834	ABC-type amino acid transport/signal transduction systems, periplasmic component/domain	ET	Amino acid transport and metabolism; signal transduction mechanisms
COG0841	Cation/multidrug efflux pump	V	Defense mechanisms
COG1006	Multisubunit Na+/H+ antiporter, MnhC subunit	P	Inorganic ion transport and metabolism
COG1009	NADH:ubiquinone oxidoreductase subunit 5 (chain L)/Multisubunit Na+/H+ antiporter, MnhA subunit	CP	Energy production and conversion; inorganic ion transport and metabolism
COG1012	NAD-dependent aldehyde dehydrogenases	C	Energy production and conversion
COG1020	Non-ribosomal peptide synthetase modules and related proteins	Q	Secondary metabolites biosynthesis, transport, and catabolism
COG1021	Peptide arylation enzymes	Q	Secondary metabolites biosynthesis, transport, and catabolism
COG1169	Isochorismate synthase	HQ	Coenzyme transport and metabolism; secondary metabolites biosynthesis, transport, and catabolism
COG1173	ABC-type dipeptide/oligopeptide/nickel transport systems, permease components	EP	Amino acid transport and metabolism; inorganic ion transport and metabolism
COG1320	Multisubunit Na+/H+ antiporter, MnhG subunit	P	Inorganic ion transport and metabolism
COG1404	Subtilisin-like serine proteases	O	Posttranslational modification, protein turnover, and chaperones
COG1523	Type II secretory pathway, pullulanase PulA and related glycosidases	G	Carbohydrate transport and metabolism
COG1535	Isochorismate hydrolase	Q	Secondary metabolites biosynthesis, transport, and catabolism
COG1733	Predicted transcriptional regulators	K	Transcription
COG1863	Multisubunit Na+/H+ antiporter, MnhE subunit	P	Inorganic ion transport and metabolism
COG2175	Probable taurine catabolism dioxygenase	Q	Secondary metabolites biosynthesis, transport, and catabolism
COG2205	Osmosensitive K+ channel histidine kinase	T	Signal transduction mechanisms
COG2212	Multisubunit Na+/H+ antiporter, MnhF subunit	P	Inorganic ion transport and metabolism
COG2375	Siderophore-interacting protein	P	Inorganic ion transport and metabolism
COG2820	Uridine phosphorylase	F	Nucleotide transport and metabolism
COG2977	Phosphopantetheinyl transferase component of siderophore synthetase	Q	Secondary metabolites biosynthesis, transport, and catabolism
COG2994	ACP:hemolysin acyltransferase (hemolysin-activating protein)	O	Posttranslational modification, protein turnover, and chaperones
COG3048	D-serine dehydratase	E	Amino acid transport and metabolism
COG3049	Penicillin V acylase and related amidases	M	Cell wall/membrane/envelope biogenesis
COG3064	Membrane protein involved in colicin uptake	M	Cell wall/membrane/envelope biogenesis
COG3201	Nicotinamide mononucleotide transporter	H	Coenzyme transport and metabolism
COG3207	Pyoverdine/dityrosine biosynthesis protein	Q	Secondary metabolites biosynthesis, transport, and catabolism
COG3284	Transcriptional activator of acetoin/glycerol metabolism	QK	Secondary metabolites biosynthesis, transport, and catabolism; transcription
COG3437	Response regulator containing a CheY-like receiver domain and an HD-GYP domain	KT	Transcription; signal transduction mechanisms
COG3479	Phenolic acid decarboxylase	Q	Secondary metabolites biosynthesis, transport, and catabolism
COG3527	Alpha-acetolactate decarboxylase	Q	Secondary metabolites biosynthesis, transport, and catabolism
COG4592	ABC-type Fe2+-enterobactin transport system, periplasmic component	P	Inorganic ion transport and metabolism
COG4774	Outer membrane receptor for monomeric catechols	P	Inorganic ion transport and metabolism
COG4779	ABC-type enterobactin transport system, permease component	P	Inorganic ion transport and metabolism
COG4935	Regulatory P domain of the subtilisin-like proprotein convertases and other proteases	O	Posttranslational modification, protein turnover, and chaperones
COG5183	Protein involved in mRNA turnover and stability	A	RNA processing and modification

[a] Functions are categorized based on the Clusters of Orthologous Groups (COG) of proteins database

[b] There are a total of 104 putative unique genes found in *V. cholerae*. Not included in this table are genes that have general function predictions only (*n* = 20), encode hypothetical proteins (*n* = 4), or have no known hits (*n* = 32)
