# Supplementary material for: Genomes of Vibrio metoecus co-isolated with Vibrio cholerae extend our understanding of differences between these closely related species
Source: Gut Pathog. 2022 Nov 20;14:42. doi: 10.1186/s13099-022-00516-x (PMC9677704; doi:10.1186/s13099-022-00516-x)
Supplement: Supplementary file 4 — Additional file 4: Presence/absence map of virulence factors in V. cholerae and V. metoecus. [file 13099_2022_516_MOESM4_ESM.pdf]

Additional file 4. Presence/absence map of virulence factors[a] in *V. cholerae* and *V. metoecus*

Virulence factors	Related genes	Reference locus tags[b]	*V. cholerae*																	*V. metoecus*																
			OYP1G01	OYP2A12	OYP2E01	OYP3B05	OYP3F10	OYP4B01	OYP4C07	OYP4G08	OYP4H06	OYP4H11	OYP6D06	OYP6E07	OYP6F08	OYP6F10	OYP7C09	OYP8C06	OYP8F12	OP3H	OYP4D01	OYP4E03	OYP5B04	OYP5B06	OYP5H08	OYP8G05	OYP8G09	OYP8G12	OYP8H05	OYP9B03	OYP9B09	OYP9C12	OYP9D03	OYP9D09	OYP9E03	OYP9E10
Adherence																																				
Accessory colonization factor	*acfA*	VC0844																																		
	acfB	VC0840																																		
	acfC	VC0841																																		
	acfD	VC0845	■	■	■			■		■	■	■	■	■	■	■	■	■	■																	
Mannose-sensitive hemagglutinin (MSHA type IV pilus)	*mshH*	VC0398	■	■	■	■	■	■	■	■	■	■	■	■	■	■	■	■	■	■	■	■	■	■	■	■	■	■	■	■	■	■	■	■	■	■
	mshI	VC0399	■	■	■	■	■	■	■	■	■	■	■	■	■	■	■	■	■	■	■	■	■	■	■	■	■	■	■	■	■	■	■	■	■	■
	mshJ	VC0400	■	■	■	■	■	■	■	■	■	■	■	■	■	■	■	■	■	■	■	■	■	■	■	■	■	■	■	■	■	■	■	■	■	■
	mshK	VC0401	■	■	■	■	■	■	■	■	■	■	■	■	■	■	■	■	■	■	■	■	■	■	■	■	■	■	■	■	■	■	■	■	■	■
	mshL	VC0402	■	■	■	■	■	■	■	■	■	■	■	■	■	■	■	■	■	■	■	■	■	■	■	■	■	■	■	■	■	■	■	■	■	■
	mshM	VC0403	■	■	■	■	■	■	■	■	■	■	■	■	■	■	■	■	■	■	■	■	■	■	■	■	■	■	■	■	■	■	■	■	■	■
	mshN	VC0404	■	■	■	■	■	■	■	■	■	■	■	■	■	■	■	■	■	■	■	■	■	■	■	■	■	■	■	■	■	■	■	■	■	■
	mshE	VC0405	■	■	■	■	■	■	■	■	■	■	■	■	■	■	■	■	■	■	■	■	■	■	■	■	■	■	■	■	■	■	■	■	■	■
	mshG	VC0406	■	■	■	■	■	■	■	■	■	■	■	■	■	■	■	■	■	■	■	■	■	■	■	■	■	■	■	■	■	■	■	■	■	■
	mshF	VC0407	■	■	■	■	■	■	■	■	■	■	■	■	■	■	■	■	■	■	■	■	■	■	■	■	■	■	■	■	■	■	■	■	■	■
	mshB	VC0408	■	■	■	■	■	■	■	■	■	■	■	■	■	■	■	■	■	■	■	■	■	■	■	■	■	■	■	■	■	■	■	■	■	■
	mshA	VC0409	■	■	■	■	■	■	■	■	■	■	■	■	■	■	■	■	■	■	■		■	■	■	■	■	■	■	■	■	■	■		■	■
	mshC	VC0410	■	■	■	■	■	■	■	■	■	■	■	■	■	■	■	■	■		■		■	■									■			
	mshD	VC0411	■	■	■	■	■	■	■	■	■	■	■	■	■	■	■	■	■	■	■	■	■	■	■	■	■	■	■	■	■	■	■	■	■	■
Toxin-coregulated pilus (type IVB pilus)	*tcpI*	VC0825																																		
	tcpP	VC0826																																		
	tcpH	VC0827																																		
	tcpA	VC0828																																		
	tcpB	VC0829																																		
	tcpQ	VC0830																																		
	tcpC	VC0831																																		
	tcpR	VC0832																																		
	tcpD	VC0833																																		
	tcpS	VC0834																																		
	tcpT	VC0835																																		
	tcpE	VC0836																																		
	tcpF	VC0837																																		
	tcpN/toxT	VC0838																																		
	tcpJ	VC0839																																		
Type IV pilus	*pilA*	VC2423																																		
	pilB	VC2424	■	■	■	■	■	■	■	■	■	■	■	■	■	■	■	■	■	■	■	■	■	■	■	■	■	■	■	■	■	■	■	■	■	■
	pilC	VC2425	■	■	■	■	■	■	■	■	■	■	■	■	■	■	■	■	■	■	■	■	■	■	■	■	■	■	■	■	■	■	■	■	■	■
	pilD	VC2426	■	■	■	■	■	■	■	■	■	■	■	■	■	■	■	■	■	■	■	■	■	■	■	■	■	■	■	■	■	■	■	■	■	■
Antiphagocytosis																																				
Capsular polysaccharide	*wza*	VF0162			■		■	■			■	■			■			■	■																	
	hp1	VV1_0785																																		
	wzb	VF0164			■		■	■			■	■			■			■	■																	
	wzc	VF0165			■		■	■			■	■			■			■	■																	
	wecA	VF0180			■		■	■			■	■			■																■					
	wecC	VC0918	■	■	■	■	■	■	■	■	■	■	■	■	■	■	■	■	■	■	■	■	■	■	■	■	■	■	■	■	■	■	■	■	■	■
	wbjD/wecB	VC0917	■	■	■	■	■	■	■	■	■	■	■	■	■	■	■	■	■	■	■	■	■	■	■	■	■	■	■	■	■	■	■	■	■	■
	wbuB	VV1_0778																																		
	wcaJ	VV0361	■																							■		■						■			
	wbfB	VF0156			■		■	■		■	■	■	■		■			■	■																	
	wbfC	VF0158																																		
	wbfT	VF0195			■	■	■	■	■	■	■	■	■	■	■	■	■	■	■	■	■		■	■	■		■									
	wbfU	VC0263			■	■	■	■	■	■	■	■	■	■	■	■	■	■	■	■	■		■	■	■		■									
	wbfY	VC0260	■	■	■	■	■	■	■	■	■	■	■	■	■	■	■	■	■	■	■	■	■	■	■	■	■	■	■	■		■	■	■	■	■
	wbfV/wcvB	VV1_0774	■	■	■	■	■	■	■	■	■	■	■	■	■	■	■	■	■	■	■	■	■	■	■	■	■	■	■	■	■	■	■	■	■	■
	rmlB	VF0166			■	■			■			■		■	■	■	■	■	■	■	■		■	■	■		■				■					
	rmlA	VF0167			■	■			■			■		■	■	■	■	■	■	■	■		■	■	■		■				■					
	rmlD	VV0302			■							■			■	■	■														■					
	rmlC	VF0168			■							■			■	■	■														■					
	cpsA	VC0934	■	■	■	■	■	■	■	■	■	■	■	■	■	■	■	■	■	■	■	■	■	■	■	■	■	■	■	■	■	■	■	■	■	■
	cpsB	VC0935	■	■	■	■	■	■	■	■	■	■	■	■	■	■	■	■	■	■	■	■	■	■	■	■	■	■	■	■	■	■	■	■	■	■
	cpsC	VC0936	■	■	■	■	■	■	■	■	■	■	■	■	■	■	■	■	■	■	■	■	■	■	■	■	■	■	■	■	■	■	■	■	■	■
	cpsD	VC0937	■	■	■	■	■	■	■	■	■	■	■	■	■	■	■	■	■	■	■	■	■	■	■	■	■	■	■	■	■	■	■	■	■	■
	cpsE	VPA1407																																		
	cpsF	VC0927	■	■	■	■	■	■	■	■	■	■	■	■	■	■	■	■	■	■	■	■	■	■	■	■	■	■	■	■	■	■	■	■	■	■
	cpsG	VPA1409																																		
	cpsH	VPA1410																																		
	cpsI	VPA1411																																		

Virulence factors	Related genes	Reference locus tags[b]	*V. cholerae*																	*V. metoecus*																
			OYP1G01	OYP2A12	OYP2E01	OYP3B05	OYP3F10	OYP4B01	OYP4C07	OYP4G08	OYP4H06	OYP4H11	OYP6D06	OYP6E07	OYP6F08	OYP6F10	OYP7C09	OYP8C06	OYP8F12	OP3H	OYP4D01	OYP4E03	OYP5B04	OYP5B06	OYP5H08	OYP8G05	OYP8G09	OYP8G12	OYP8H05	OYP9B03	OYP9B09	OYP9C12	OYP9D03	OYP9D09	OYP9E03	OYP9E10
Capsular polysaccharide	*cpsJ*	VPA1412																																		
Chemotaxis and motility																																				
Flagella	*flaC*	VC2187	■	■	■	■	■	■	■	■	■	■	■	■	■	■	■	■	■	■	■	■	■	■	■	■	■	■	■	■	■	■	■	■	■	■
	flaA	VC2188	■	■	■	■	■	■	■	■	■	■	■	■	■	■	■	■	■	■	■	■	■	■	■	■	■	■	■	■	■	■	■	■	■	■
	flgL	VC2190	■	■	■	■	■	■	■	■	■	■	■	■	■	■	■	■	■	■	■	■	■	■	■	■	■	■	■	■	■	■	■	■	■	■
	flgK	VC2191	■	■	■	■	■	■	■	■	■	■	■	■	■	■	■	■	■	■	■	■	■	■	■	■	■	■	■	■	■	■	■	■	■	■
	flgJ	VC2192	■	■	■	■	■	■	■	■	■	■	■	■	■	■	■	■	■	■	■	■	■	■	■	■	■	■	■	■	■	■	■	■	■	■
	flgI	VC2193	■	■	■	■	■	■	■	■	■	■	■	■	■	■	■	■	■	■	■	■	■	■	■	■	■	■	■	■	■	■	■	■	■	■
	flgH	VC2194	■	■	■	■	■	■	■	■	■	■	■	■	■	■	■	■	■	■	■	■	■	■	■	■	■	■	■	■	■	■	■	■	■	■
	flgG	VC2195	■	■	■	■	■	■	■	■	■	■	■	■	■	■	■	■	■	■	■	■	■	■	■	■	■	■	■	■	■	■	■	■	■	■
	flgF	VC2196	■	■	■	■	■	■	■	■	■	■	■	■	■	■	■	■	■	■	■	■	■	■	■	■	■	■	■	■	■	■	■	■	■	■
	flgE	VC2197	■	■	■	■	■	■	■	■	■	■	■	■	■	■	■	■	■	■	■	■	■	■	■	■	■	■	■	■	■	■	■	■	■	■
	flgD	VC2198	■	■	■	■	■	■	■	■	■	■	■	■	■	■	■	■	■	■	■	■	■	■	■	■	■	■	■	■	■	■	■	■	■	■
	flgC	VC2199	■	■	■	■	■	■	■	■	■	■	■	■	■	■	■	■	■	■	■	■	■	■	■	■	■	■	■	■	■	■	■	■	■	■
	flgB	VC2200	■	■	■	■	■	■	■	■	■	■	■	■	■	■	■	■	■	■	■	■	■	■	■	■	■	■	■	■	■	■	■	■	■	■
	cheR	VC2201	■	■	■	■	■	■	■	■	■	■	■	■	■	■	■	■	■	■	■	■	■	■	■	■	■	■	■	■	■	■	■	■	■	■
	cheV	VC2202	■	■	■	■	■	■	■	■	■	■	■	■	■	■	■	■	■	■	■	■	■	■	■	■	■	■	■	■	■	■	■	■	■	■
	flgA	VC2203	■	■	■	■	■	■	■	■	■	■	■	■	■	■	■	■	■	■	■	■	■	■	■	■	■	■	■	■	■	■	■	■	■	■
	flgM	VC2204	■	■	■	■	■	■	■	■	■	■	■	■	■	■	■	■	■	■	■	■	■	■	■	■	■	■	■	■	■	■	■	■	■	■
	flgN	VC2205	■	■	■	■	■	■	■	■	■	■	■	■	■	■	■	■	■	■	■	■	■	■	■	■	■	■	■	■	■	■	■	■	■	■
	flgP	VC2206	■	■	■	■	■	■	■	■	■	■	■	■	■	■	■	■	■	■	■	■	■	■	■	■	■	■	■	■	■	■	■	■	■	■
	flgO	VC2207	■	■	■	■	■	■	■	■	■	■	■	■	■	■	■	■	■	■	■	■	■	■	■	■	■	■	■	■	■	■	■	■	■	■
	flgT	VC2208	■	■	■	■	■	■	■	■	■	■	■	■	■	■	■	■	■	■	■	■	■	■	■	■	■	■	■	■	■	■	■	■	■	■
	flhB	VC2120	■	■	■	■	■	■	■	■	■	■	■	■	■	■	■	■	■	■	■	■	■	■	■	■	■	■	■	■	■	■	■	■	■	■
	fliR	VC2121	■	■	■	■	■		■	■		■	■	■	■	■	■	■	■	■	■	■	■	■	■	■	■	■	■	■	■	■	■	■	■	■
	fliQ	VC2122	■	■	■	■	■	■	■	■	■	■	■	■	■	■	■	■	■	■	■	■	■	■	■	■	■	■	■	■	■	■	■	■	■	■
	fliP	VC2123	■	■		■	■	■	■	■	■	■	■	■	■	■	■	■	■	■	■	■	■	■	■	■	■	■	■	■	■	■	■	■	■	■
	fliO	VC2124	■	■		■	■	■	■	■	■	■	■	■	■	■	■	■	■	■	■	■	■	■	■	■	■	■	■	■	■	■	■	■	■	■
	fliN	VC2125	■	■		■	■	■	■	■	■	■	■	■	■	■	■	■	■	■	■	■	■	■	■	■	■	■	■	■	■	■	■	■	■	■
	filM	VC2126	■	■		■	■	■	■	■	■	■	■	■	■	■	■	■	■	■	■	■	■	■	■	■	■	■	■	■	■	■	■	■	■	■
	fliL	VC2127	■	■	■	■	■	■	■	■	■	■	■	■	■	■	■	■	■	■	■	■	■	■	■	■	■	■	■	■	■	■	■	■	■	■
	fliK	VC2128	■	■	■	■	■	■	■	■	■	■	■	■	■	■	■	■	■	■	■	■	■	■	■	■	■	■	■	■	■	■	■	■	■	■
	fliJ	VC2129	■	■	■	■	■	■	■	■	■	■	■	■	■	■	■	■	■	■	■	■	■	■	■	■	■	■	■	■	■	■	■	■	■	■
	fliI	VC2130	■	■	■	■	■	■	■	■	■	■	■	■	■	■	■	■	■	■	■	■	■	■	■	■	■	■	■	■	■	■	■	■	■	■
	fliH	VC2131	■	■	■	■	■	■	■	■	■	■	■	■	■	■	■	■	■	■	■	■	■	■	■	■	■	■	■	■	■	■	■	■	■	■
	fliG	VC2132	■	■	■	■	■	■	■	■	■	■	■	■	■	■	■	■	■	■	■	■	■	■	■	■	■	■	■	■	■	■	■	■	■	■
	fliF	VC2133	■	■		■	■	■	■	■	■	■	■	■	■	■	■	■	■	■	■	■	■	■	■	■	■	■	■	■	■	■	■	■	■	■
	fliE	VC2134	■	■	■	■	■	■	■	■	■	■	■	■	■	■	■	■	■	■	■	■	■	■	■	■	■	■	■	■	■	■	■	■	■	■
	flrC	VC2135	■	■	■	■	■	■	■	■	■	■	■	■	■	■	■	■	■	■	■	■	■	■	■	■	■	■	■	■	■	■	■	■	■	■
	flrB	VC2136	■	■	■	■	■	■	■	■	■	■	■	■	■	■	■	■	■	■	■	■	■	■	■	■	■	■	■	■	■	■	■	■	■	■
	flrA	VC2137	■	■		■	■	■	■	■	■	■	■	■	■	■	■	■	■	■	■	■	■	■	■	■	■	■	■	■	■	■	■	■	■	■
	fliS	VC2138	■	■	■	■	■	■	■	■	■	■	■	■	■	■	■	■	■	■	■	■	■	■	■	■	■	■	■	■	■	■	■	■	■	■
	flaI	VC2139	■	■	■	■	■	■	■	■	■	■	■	■	■	■	■	■	■	■	■	■	■	■	■	■	■	■	■	■	■	■	■	■	■	■
	fliD	VC2140	■	■	■	■	■	■	■	■	■	■	■	■	■	■	■	■	■	■	■	■	■	■	■	■	■	■	■	■	■	■	■	■	■	■
	flaG	VC2141	■	■	■	■	■	■	■	■	■	■	■	■	■	■	■	■	■	■	■	■	■	■	■	■	■	■	■	■	■	■	■	■	■	■
	flaB	VC2142	■	■	■	■	■	■	■	■	■	■	■	■	■	■	■	■	■	■	■	■	■	■	■	■	■	■	■	■	■	■	■	■	■	■
	flaD	VC2143	■	■	■	■	■	■	■	■	■	■	■	■	■	■	■	■	■	■	■	■	■	■	■	■	■	■	■	■	■	■	■	■	■	■
	flaE	VC2144	■	■	■	■	■	■	■	■	■	■	■	■	■	■	■	■	■	■	■	■	■	■	■	■	■	■	■	■	■	■	■	■	■	■
	cheW	VC2059	■	■	■	■	■	■	■	■	■	■	■	■	■	■	■	■	■	■	■	■	■	■	■	■	■	■	■	■	■	■	■	■	■	■
	cheB	VC2062	■	■	■	■	■	■	■	■	■	■	■	■	■	■	■	■	■	■	■	■	■	■	■	■	■	■	■	■	■	■	■	■	■	■
	cheA	VC2063	■	■	■	■	■	■	■	■	■	■	■	■	■	■	■	■	■	■	■	■	■	■	■	■	■	■	■	■	■	■	■	■	■	■
	cheZ	VC2064	■	■	■	■	■	■	■	■	■	■	■	■	■	■	■	■	■	■	■	■	■	■	■	■	■	■	■	■	■	■	■	■	■	■
	cheY	VC2065	■	■	■	■	■	■	■	■	■	■	■	■	■	■	■	■	■	■	■	■	■	■	■	■	■	■	■	■	■	■	■	■	■	■
	fliA	VC2066	■	■	■	■	■	■	■	■	■	■	■	■	■	■	■	■	■	■	■	■	■	■	■	■	■	■	■	■	■	■	■	■	■	■
	flhG	VC2067	■	■	■	■	■	■	■	■	■	■	■	■	■	■	■	■	■	■	■	■	■	■	■	■	■	■	■	■	■	■	■	■	■	■
	flhF	VC2068	■	■	■	■	■	■	■	■	■	■	■	■	■	■	■	■	■	■	■	■	■	■	■	■	■	■	■	■	■	■	■	■	■	
	flhA	VC2069	■	■	■	■	■	■	■	■	■	■	■	■	■	■	■	■	■	■	■	■	■	■	■	■	■	■	■	■	■	■	■	■	■	■
	motA	VC0892	■	■	■	■	■	■	■	■	■	■	■	■	■	■	■	■	■	■	■	■	■	■	■	■	■	■	■	■	■	■	■	■	■	■
	motB	VC0893	■	■	■	■	■	■	■	■	■	■	■	■	■	■	■	■	■	■	■	■	■	■	■	■	■	■	■	■	■	■	■	■	■	■
	motY	VC1008	■	■	■	■	■	■	■	■	■	■	■	■	■	■	■	■	■	■	■	■	■	■	■	■	■	■	■	■	■	■	■	■	■	■
	motX	VC2601	■	■	■	■	■	■	■	■	■	■	■	■	■	■	■	■	■	■	■	■	■	■	■	■	■	■	■	■	■	■	■	■	■	■
Exoenzyme																																				
Metalloproteases	*hap/vvp*	VCA0865	■	■	■	■	■	■	■	■	■	■	■	■	■	■	■	■	■	■	■	■	■	■	■	■	■	■	■	■	■	■	■	■	■	■
Neuraminidase	*nanH*	VC1784					■	■		■	■							■	■	■	■	■	■	■	■		■	■	■	■		■	■	■	■	■
Iron uptake																																				
Enterobactin receptors	*vctA*	VC0395_0996	■	■	■	■	■	■	■	■	■	■	■	■	■	■	■	■	■	■	■		■	■	■	■	■	■			■	■				
	irgA	VC0475	■	■	■	■	■	■	■	■	■	■	■	■	■	■	■	■	■	■	■	■	■	■	■	■	■	■	■	■	■	■	■	■	■	■

Virulence factors	Related genes	Reference locus tags[b]	*V. cholerae*																	*V. metoecus*																
			OYP1G01	OYP2A12	OYP2E01	OYP3B05	OYP3F10	OYP4B01	OYP4C07	OYP4G08	OYP4H06	OYP4H11	OYP6D06	OYP6E07	OYP6F08	OYP6F10	OYP7C09	OYP8C06	OYP8F12	OP3H	OYP4D01	OYP4E03	OYP5B04	OYP5B06	OYP5H08	OYP8G05	OYP8G09	OYP8G12	OYP8H05	OYP9B03	OYP9B09	OYP9C12	OYP9D03	OYP9D09	OYP9E03	OYP9E10
Heme receptors	*hutA*	VCA0576																																		
	hutR	VCA0064																																		
	hasR	VCA0625																																		
Periplasmic binding protein-dependent ABC transport systems	*viuP*	VC0776																																		
	viuD	VC0777																																		
	viuG	VC0778																																		
	viuC	VC0779																																		
	vctP	VCA0227																																		
	vctD	VCA0228																																		
	vctG	VCA0229																																		
	vctC	VCA0230																																		
Vibriobactin biosynthesis	*vibB*	VC0771																																		
	vibE	VC0772																																		
	vibC	VC0773																																		
	vibA	VC0774																																		
	vibH	VC0775																																		
	vibD	VC0780																																		
	vibF	VC2209																																		
Vibriobactin utilization	*viuB*	VC2210																																		
	viuA	VC2211																																		
Quorum sensing																																				
Autoinducer-2	*luxS*	VC0557																																		
Cholerae autoinducer-1	*cqsA*	VCA0523																																		
Secretion system																																				
EPS type II secretion system	*epsN*	VC2723																																		
	epsM	VC2724																																		
	epsL	VC2725																																		
	epsK	VC2726																																		
	epsJ	VC2727																																		
	epsI	VC2728																																		
	epsH	VC2729																																		
	epsG	VC2730																																		
	epsF	VC2731																																		
	epsE	VC2732																																		
	gspD	VC2733																																		
	epsC	VC2734																																		
Type III secretion system-1	*vopD*	VP1656																																		
	vopB	VP1657																																		
	vcrH	VP1658																																		
	vcrV	VP1659																																		
	vcrG	VP1660																																		
	vcrR	VP1661																																		
	vcrD	VP1662																																		
	vscY	VP1663																																		
	vscX	VP1664																																		
	sycN	VP1665																																		
	tyeA	VP1666																																		
	vopN	VP1667																																		
	vscN	VP1668																																		
	vscO	VP1669																																		
	vscP	VP1670																																		
	vscQ	VP1671																																		
	vscR	VP1672																																		
	vscS	VP1673																																		
	vscT	VP1674																																		
	vscU	VP1675																																		
	vecA	VP1682																																		
	vscL	VP1688																																		
	vscK	VP1689																																		
	vscJ	VP1690																																		
	vscI	VP1691																																		
	vscH	VP1692																																		
	vscG	VP1693																																		
	vscF	VP1694																																		
	vscD	VP1695																																		
	vscC	VP1696																																		
	vscB	VP1697																																		

Virulence factors	Related genes	Reference locus tags[b]	*V. cholerae*																	*V. metoecus*																
			OYP1G01	OYP2A12	OYP2E01	OYP3B05	OYP3F10	OYP4B01	OYP4C07	OYP4G08	OYP4H06	OYP4H11	OYP6D06	OYP6E07	OYP6F08	OYP6F10	OYP7C09	OYP8C06	OYP8F12	OP3H	OYP4D01	OYP4E03	OYP5B04	OYP5B06	OYP5H08	OYP8G05	OYP8G09	OYP8G12	OYP8H05	OYP9B03	OYP9B09	OYP9C12	OYP9D03	OYP9D09	OYP9E03	OYP9E10
Type III secretion system-1	*vscA*	VP1698																																		
	virF	VP1699																																		
	virG	VP1700																																		
	vxsC	VP1701																																		
Type III secretion system-1 secreted effectors	*vopS*	VP1686																																		
	vopR	VP1683																																		
	vopQ	VP1680																																		
	-	VPA0450																																		
Type III secretion system-2	*ospC2*	VPA1331																																		
	vtrA	VPA1332																																		
	vscS2	VPA1335																																		
	-	VPA1337																																		
	vscN2	VPA1338																																		
	vscC2	VPA1339																																		
	-	VPA1340																																		
	vscT2	VPA1341																																		
	vscR2	VPA1342																																		
	vscQ2	VPA1349																																		
	-	VPA1350																																		
	-	VPA1351																																		
	-	VPA1352																																		
	-	VPA1353																																		
	vscU2	VPA1354																																		
	vcrD2	VPA1355																																		
	vopD2	VPA1361																																		
	vopB2	VPA1362																																		
	-	VPA1363																																		
	vscJ2	VPA1367																																		
	ospB2	VPA1380																																		
Type III secretion system-2 secreted effectors	*vopC*	VPA1321																																		
	vopT	VPA1327																																		
	vopZ	VPA1336																																		
	vopA	VPA1346																																		
	vopL	VPA1370																																		
Virulence associated secretion effector proteins (type VI secretion system)	*hcp-1*	VC1415																																		
	hcp-2	VCA0017																																		
	vgrG-1	VC1416																																		
	vgrG-2	VCA0018																																		
	vgrG-3	VCA0123																																		
Virulence associated secretion genes (type VI secretion system)	*vipA/mglA*	VCA0107																																		
	vipB/mglB	VCA0108																																		
	-	VCA0109																																		
	vasA	VCA0110																																		
	vasB	VCA0111																																		
	vasC	VCA0112																																		
	vasD	VCA0113																																		
	vasE	VCA0114																																		
	vasF	VCA0115																																		
	vasG	VCA0116																																		
	vasH	VCA0117																																		
	vasI	VCA0118																																		
	vasJ	VCA0119																																		
	vasK	VCA0120																																		
	vasL	VCA0121																																		
	-	VCA0122																																		
Toxin																																				
Accessory cholera enterotoxin	*ace*	VC1459																																		
Cholera toxin	*ctxA*	VC1457																																		
	ctxB	VC1456																																		
RTX toxin	*rtxD*	VC1447																																		
	rtxB	VC1448																																		
	rtxC	VC1450																																		
	rtxA	VC1451																																		
Thermolabile hemolysin	*tlh*	VCA0218																																		
Vibrio cholerae cytolysin	*hlyA*	VCA0219																																		
Zona occludens toxin	*zot*	VC1458																																		
Hemolysin/cytolysin	*vvhA*	VV2_0404																																		

Virulence factors	Related genes	Reference locus tags[b]	***V. cholerae***																	***V. metoecus***																
			**OYP1G01**	**OYP2A12**	**OYP2E01**	**OYP3B05**	**OYP3F10**	**OYP4B01**	**OYP4C07**	**OYP4G08**	**OYP4H06**	**OYP4H11**	**OYP6D06**	**OYP6E07**	**OYP6F08**	**OYP6F10**	**OYP7C09**	**OYP8C06**	**OYP8F12**	**OP3H**	**OYP4D01**	**OYP4E03**	**OYP5B04**	**OYP5B06**	**OYP5H08**	**OYP8G05**	**OYP8G09**	**OYP8G12**	**OYP8H05**	**OYP9B03**	**OYP9B09**	**OYP9C12**	**OYP9D03**	**OYP9D09**	**OYP9E03**	**OYP9E10**
Thermostable direct hemolysin	*tdhA*	VPA1314																																		
	tdhS	VPA1378																																		

[a] Virulence factors for the genus *Vibrio* were obtained from the VFDB (http://www.mgc.ac.cn/cgi-bin/VFs/genus.cgi?Genus=Vibrio). Colored squares represent BLAST score ratios of at least 0.3 against reference genes (i.e., a homologues of genes are present); white squares represent absence of genes

[b] Locus tags indicate the species and strains for the reference sequences. VC: *V. cholerae* N16961; VC0395: *V. cholerae* O395; VF: *Vibrio fischeri* ES114 (*Aliivibrio fischeri*); VP: *Vibrio parahaemolyticus* RIMD 210633; VV: *Vibrio vulnificus* CMCP6
