# Supplementary material for: Genomes of Vibrio metoecus co-isolated with Vibrio cholerae extend our understanding of differences between these closely related species
Source: Gut Pathog. 2022 Nov 20;14:42. doi: 10.1186/s13099-022-00516-x (PMC9677704; doi:10.1186/s13099-022-00516-x)
Supplement: Supplementary file 5 — Additional file 5: Presence/absence map of genes of the Vibrio pathogenicity island – 2 in V. cholerae and V. metoecus. [file 13099_2022_516_MOESM5_ESM.pdf]

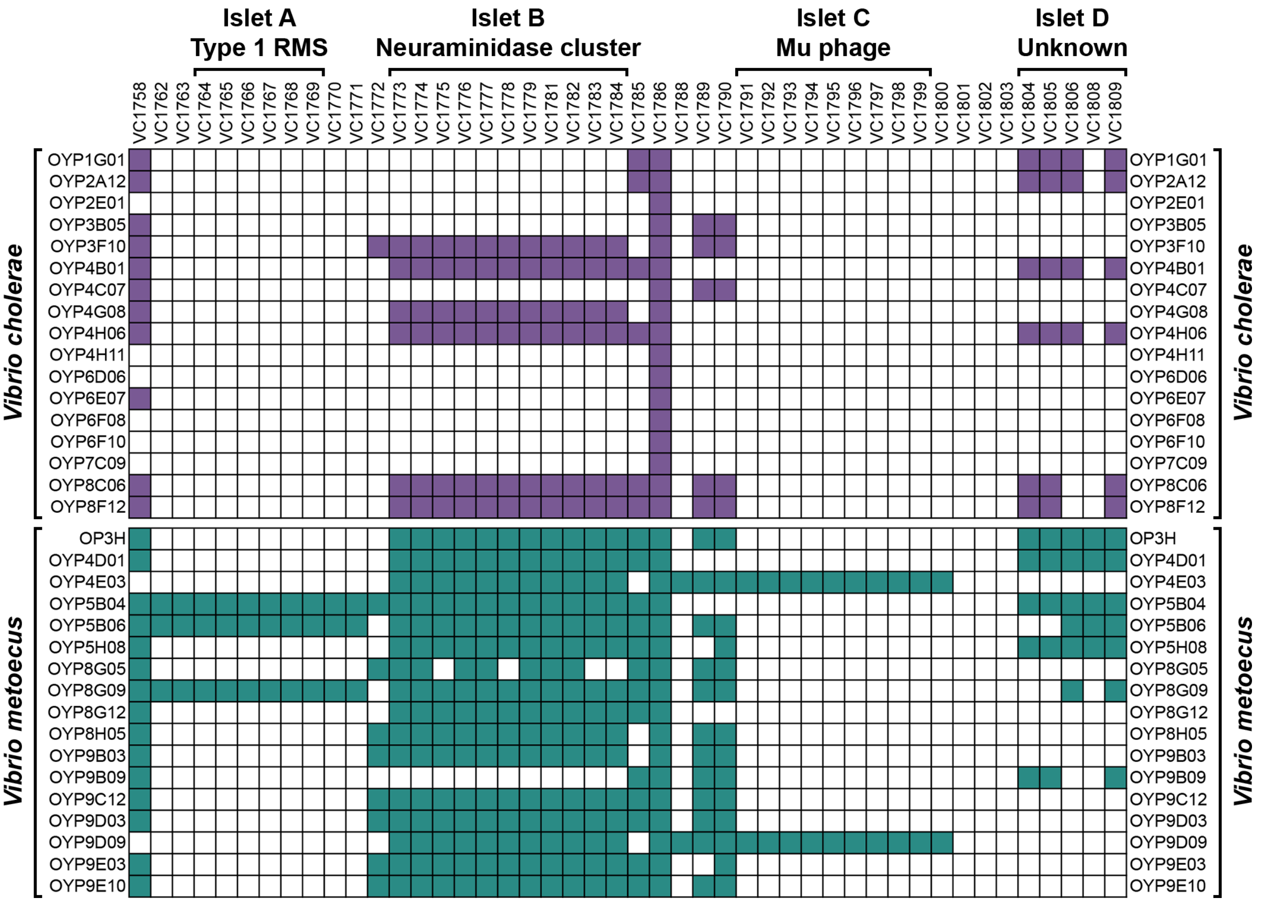

Additional file 5. Presence/absence map of genes of the *Vibrio* pathogenicity island – 2 (VPI-2) in *V. cholerae* and *V. metoecus*. Each column is a gene from the VPI-2 of the reference genome *V. cholerae* N16961, labeled with their corresponding locus tags. Colored squares represent BLAST score ratios of at least 0.3 against reference genes (i.e., homologues of genes are present); white squares represent absence of genes. The four major sections (islets) of VPI-2 are indicated. RMS: restriction-modification system.
