# Supplementary material for: Genomes of Vibrio metoecus co-isolated with Vibrio cholerae extend our understanding of differences between these closely related species
Source: Gut Pathog. 2022 Nov 20;14:42. doi: 10.1186/s13099-022-00516-x (PMC9677704; doi:10.1186/s13099-022-00516-x)
Supplement: Supplementary file 6 — Additional file 6: The pan-genome of V. cholerae and V. metoecus. [file 13099_2022_516_MOESM6_ESM.pdf]

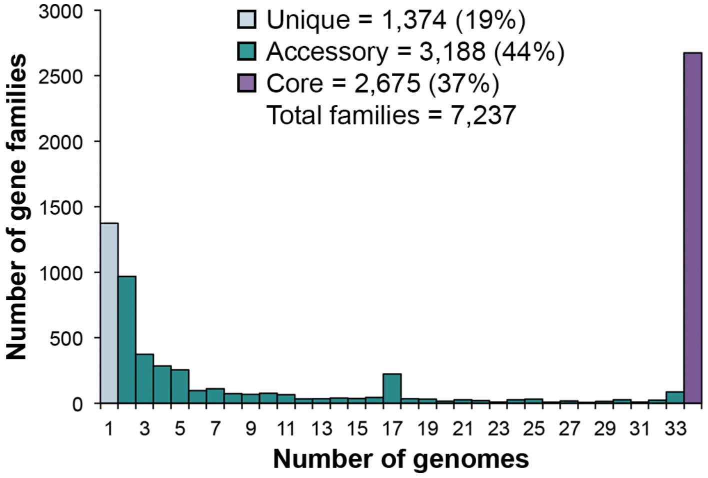

Additional file 6. The pan-genome of *V. cholerae* and *V. metoecus*. The bar graph shows the distribution of unique (present in one strain only), accessory (present in some strains), and core (100% present in all strains) gene families.
