# Supplementary material for: Genomes of Vibrio metoecus co-isolated with Vibrio cholerae extend our understanding of differences between these closely related species
Source: Gut Pathog. 2022 Nov 20;14:42. doi: 10.1186/s13099-022-00516-x (PMC9677704; doi:10.1186/s13099-022-00516-x)
Supplement: Supplementary file 7 — Additional file 7: Count of horizontal gene transfer events within the core genes of V. cholerae and V. metoecus. [file 13099_2022_516_MOESM7_ESM.pdf]

Additional file 7. Count of horizontal gene transfer (HGT) events within the core genes[a] of *V. cholerae* and *V. metoecus*

Species and strain	HGT count[b]	Percent of total
V. cholerae		
OYP6E07	42	3.07
OYP8C06	32	2.34
OYP8F12	32	2.34
OYP4G08	27	1.97
OYP1G01	24	1.75
OYP7C09	18	1.32
OYP3F10	17	1.24
OYP4H11	17	1.24
OYP6F10	17	1.24
OYP2A12	16	1.17
OYP4B01	16	1.17
OYP6D06	16	1.17
OYP4H06	15	1.10
OYP2E01	14	1.02
OYP4C07	14	1.02
OYP3B05	13	0.95
OYP6F08	11	0.80
V. cholerae total	341	24.93
V. metoecus		
OP3H	85	6.21
OYP5H08	73	5.34
OYP4D01	72	5.26
OYP4E03	67	4.90
OYP9D09	67	4.90
OYP8H05	66	4.82
OYP9B03	64	4.68
OYP5B06	63	4.61
OYP8G09	63	4.61
OYP8G12	63	4.61
OYP9B09	59	4.31
OYP5B04	58	4.24
OYP8G05	53	3.87
OYP9C12	47	3.44
OYP9D03	43	3.14
OYP9E10	43	3.14
OYP9E03	41	3.00
V. metoecus total	1,027	75.07
Total	1,368	100.00

[a] Transfer events were counted from 554 single-copy core gene phylogenetic trees, where *V. cholerae* and *V. metoecus* members cannot be partitioned into two perfect clades

[b] The values indicate the number of times a strain is a recipient of a gene transfer event, where that strain clustered with members of the other species (donor) in a tree with reliable bootstrap support (≥ 70%). Mann-Whitney-Wilcoxon test, $p < 0.001$
