# Supplementary material for: Genomes of Vibrio metoecus co-isolated with Vibrio cholerae extend our understanding of differences between these closely related species
Source: Gut Pathog. 2022 Nov 20;14:42. doi: 10.1186/s13099-022-00516-x (PMC9677704; doi:10.1186/s13099-022-00516-x)
Supplement: Supplementary file 8 — Additional file 8: Count of horizontal gene transfer events within the accessory genes of V. cholerae and V. metoecus. [file 13099_2022_516_MOESM8_ESM.pdf]

Additional file 8. Count of horizontal gene transfer (HGT) events within the accessory genes[a] of *V. cholerae* and *V. metoecus*

Species and strain	HGT count[b]	Percent of total
V. cholerae		
OYP3F10	43	5.49
OYP8C06	30	3.83
OYP8F12	28	3.58
OYP6E07	25	3.19
OYP1G01	24	3.07
OYP3B05	22	2.81
OYP4G08	22	2.81
OYP4C07	20	2.55
OYP6F08	19	2.43
OYP7C09	18	2.30
OYP2A12	17	2.17
OYP6F10	16	2.04
OYP2E01	15	1.92
OYP4B01	15	1.92
OYP4H06	14	1.79
OYP4H11	14	1.79
OYP6D06	12	1.53
V. cholerae total	354	45.21
V. metoecus		
OYP9B09	48	6.13
OP3H	32	4.09
OYP5B04	32	4.09
OYP4E03	30	3.83
OYP9D09	30	3.83
OYP8G12	28	3.58
OYP9C12	26	3.32
OYP8H05	25	3.19
OYP9B03	24	3.07
OYP5H08	23	2.94
OYP8G05	23	2.94
OYP8G09	22	2.81
OYP4D01	21	2.68
OYP9E03	20	2.55
OYP5B06	17	2.17
OYP9E10	15	1.92
OYP9D03	13	1.66
V. metoecus total	429	54.79
Total	783	100.00

[a] Transfer events were counted from 178 phylogenetic trees, where *V. cholerae* and *V. metoecus* members cannot be partitioned into two perfect clades

[b] The values indicate the number of times a strain is a recipient of a gene transfer event, where that strain clustered with members of the other species (donor) in a tree with reliable bootstrap support (≥ 70%). Equal variance *t*-test, $p = 0.114$
