# Supplementary material for: Genomes of Vibrio metoecus co-isolated with Vibrio cholerae extend our understanding of differences between these closely related species
Source: Gut Pathog. 2022 Nov 20;14:42. doi: 10.1186/s13099-022-00516-x (PMC9677704; doi:10.1186/s13099-022-00516-x)
Supplement: Supplementary file 9 — Additional file 9: Presence/absence map of genes involved in DNA uptake and restriction-modification, as well as endo- and exonucleases in V. cholerae and V. metoecus. [file 13099_2022_516_MOESM9_ESM.pdf]

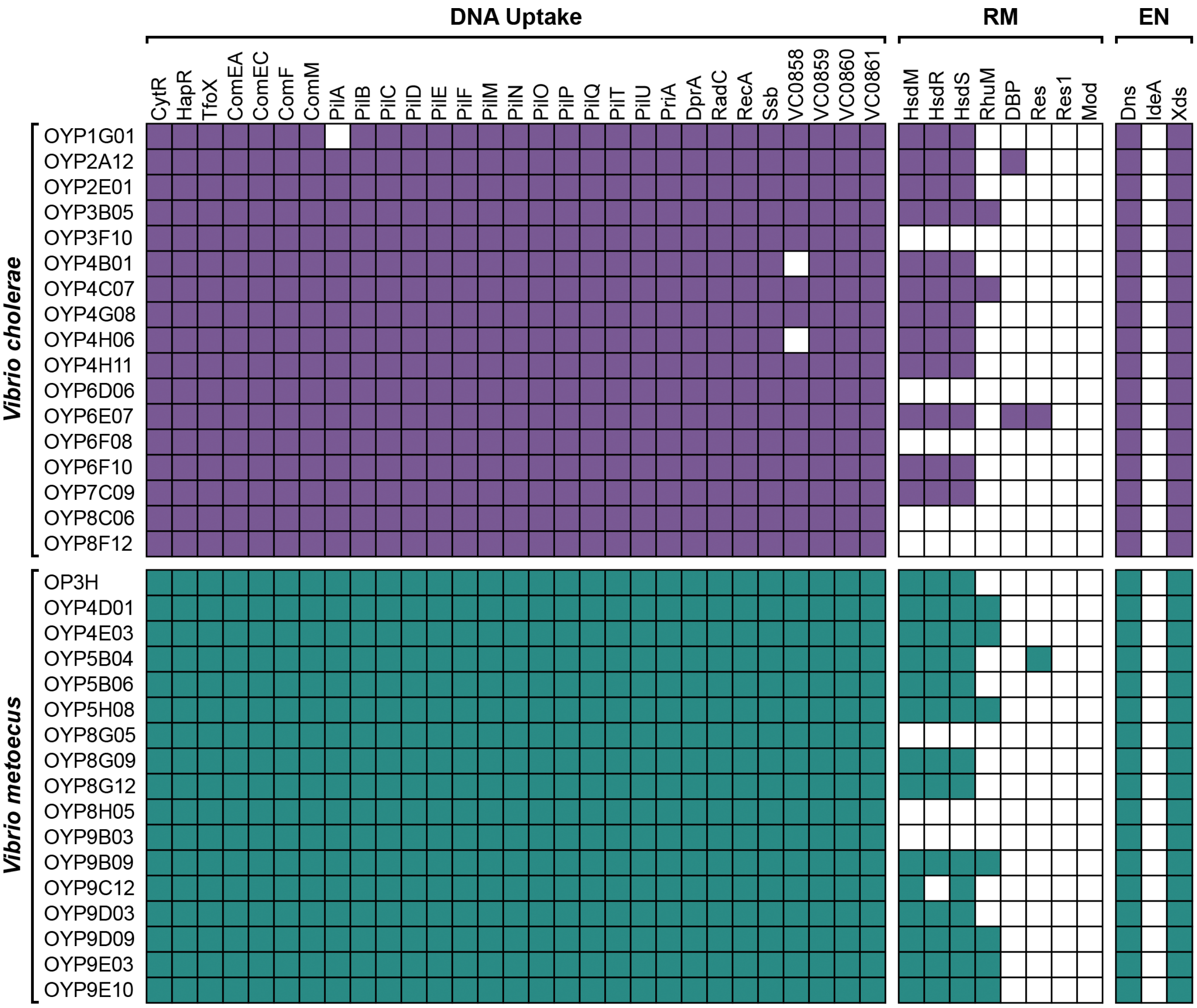

Additional file 9. Presence/absence map of genes involved in DNA uptake and restriction-modification (RM), as well as endo- and exonucleases (EN) in *V. cholerae* and *V. metoecus*. Colored squares represent the presence of genes; white squares represent the absence of genes. Presence of DNA uptake and *ideA* (EN) genes were determined by BLAST comparisons against reference genes from *V. cholerae* N16961 or 2010EL-1786, respectively, where BLAST score ratios of at least 0.3 indicate that homologues of genes are present. Presence of RM and other EN genes were obtained from RAST annotations.
